# Menstrual blood-derived endometrial stem cells alleviate neuroinflammation by modulating M1/M2 polarization in cell and rat Parkinson’s disease models

**DOI:** 10.1186/s13287-023-03330-7

**Published:** 2023-04-13

**Authors:** Han Li, Jinghui Wei, Zhigang Zhang, Junyao Li, Yaokai Ma, Ping Zhang, Juntang Lin

**Affiliations:** 1 Stem Cells and Biotherapy Engineering Research Center of Henan, National Joint Engineering Laboratory of Stem Cells and Biotherapy, School of Life Science and Technology, Xinxiang Medical University, Xinxiang, 453003 China; 2 Department of Neurology, The First Affiliated Hospital of Xinxiang Medical University, Xinxiang, 45003 China; 3 Henan Joint International Research Laboratory of Stem Cell Medicine, School of Medical Engineering, Xinxiang Medical University, Xinxiang, 453003 China

**Keywords:** Menstrual blood-derived endometrial stem cells, Parkinson’s disease, M1, M2 neuroinflammation

## Abstract

**Background:**

Neuroinflammation is closely related to the development of Parkinson's disease (PD). Because of the extensive sources, non-invasive and periodical collection method, human menstrual blood-derived endometrial stem cells (MenSCs) have been explored as a promising tool for treatment of PD. This study aimed to investigate if MenSCs could inhibit neuroinflammation in PD rats by regulating M1/M2 polarization and to excavate the underlying mechanisms.

**Methods:**

MenSCs were co-cultured with 6-OHDA-exposed microglia cell lines. Then the morphology of microglia cells and the level of inflammatory factors were assessed by immunofluorescence and qRT-PCR. After MenSCs were transplanted into the brain of PD rats, animal motor function, the expression of tyrosine hydroxylase, and the level of inflammatory factors in the cerebrospinal fluid (CSF) and serum were detected to evaluate the therapeutic potential of MenSCs. Meanwhile, the expression of M1/M2 phenotype related genes was detected by qRT-PCR. One protein array kit containing 1000 kinds of factors was used to detect the protein components in the conditioned medium of MenSCs. Finally, bioinformatic analysis was performed to analyze the function of factors secreted by MenSCs and the signal pathways involved in.

**Results:**

MenSCs could suppress 6-OHDA-induced microglia cell activation and significantly decrease inflammation in vitro. After transplantation into the brain of PD rats, MenSCs improved animal motor function, which was indicated by the increased movement distance, ambulatory episodes, exercise time on the rotarod, and less contralateral rotation. Additionally, MenSCs reduced the loss of dopaminergic neurons and down-regulated the level of pro-inflammatory factors in the CSF and serum. Moreover, q-PCR and WB results showed the transplantation of MenSCs significantly down-regulated the expression of M1 phenotype cell markers and meanwhile up-regulated the expression of M2 phenotype cell markers in the brain of PD rats. 176 biological processes including inflammatory response, negative regulation of apoptotic process, and microglial cell activation were enriched by GO-BP analysis. 58 signal pathways including PI3K/Akt and MAPK were enriched by KEGG analysis.

**Conclusions:**

In conclusion, our results provide preliminary evidence for the anti-inflammation capacity of MenSCs by regulating M1/M2 polarization. We firstly demonstrated the biological process of factors secreted by MenSCs and the signal pathways involved in using protein array and bioinformatic analysis.

**Supplementary Information:**

The online version contains supplementary material available at 10.1186/s13287-023-03330-7.

## Introduction

Although the pathogenesis of Parkinson’s disease (PD) is not completely revealed, mounting evidences prove that the dysregulation of immune system including innate and adaptive immunity plays an essential role in the pathogenesis of PD [1–3]. Microglia cells are a predominant type of resident immune cells in the brain [2, 4]. They account for about 12% of total cells and play an important role in immune surveillance and central nervous system homeostasis [4]. During development, they are involved in synaptic pruning and removing normally occurring apoptotic neurons by efferocytosis [4]. Besides, they can release low level neurotropic factors to support neurons and glia cells survival [2]. When the central nervous system is infected by pathogens or in presence of tissue damage, resting microglia cells undergo a series of changes in morphology, gene expression and function, and become activated [5]. After activation, microglia can be divided into M1 state and M2 state [5, 6]. Microglia in the M2 state can release anti-inflammatory factors, such as IL-4 and IL-13, engulf damaged neuron fragments, and promote tissue repair and neurons regeneration [6]. But when microglia cells are over-activated, they turn to the M1 state and release a large number of neuroinflammatory factors such as IL-1β, IL-6, TNF-α by activating MAPKs/NF-κB/ERK pathway and lead to neurons apoptosis [6]. Microglia cells have a close relationship with the progress of PD [7]. Excessive ROS and activated M1 type microglia cells were found in the postmortem of PD patients [2]. In 6-OHDA induced PD rats, M2 phenotype microglia increased in the first 3 days after drug treatment, then gradually decreased and shifted to M1 phenotype at later time points, when dopaminergic neurons death was manifest [8]. Therefore, prevention microglia polarization to M1 phenotype may be a target for prevention and treatment of PD.

Menstrual blood-derived endometrial stem cells (MenSCs) came to the attention of public in 2007 [9, 10]. Compared to other sources of MSCs, menstrual blood could be obtained periodicity, non-invasively, and without trauma [11]. According to our previous study, 30–50 ml menstrual blood could be collected during menstruation and 5 ml sample could expand 100 million passage 3 MenSCs, which is enough for clinical treatment [11]. As a kind of attractive seed cell for stem cell-based therapies, they have been used for treatment of various inflammatory related diseases, such as lung injury [12], colitis [13], pulmonary fibrosis [14], experimental arthritis [15], liver failure [16], autoimmune encephalomyelitis [17], stroke [18], and severe COVID‑19 patients [19] by immunomodulation function. However, whether MenSCs could be feasible to alleviate neuroinflammation by modulating microglia polarization in PD remain unclear, and which factors released by MenSCs contributed to their therapeutic function in PD needs to be clarified.

In this study, we investigated the anti-inflammatory function of MenSCs on BV2 and HAPI cell lines by inhibiting excessive activation of microglia cells. In addition, we explored the therapeutic effect of MenSCs on a PD rat model, mainly focused on anti-inflammation by modulating M1/M2 polarization. Furthermore, we elucidated possible neuroregulatory molecules that mediate the neuroprotective and immunomodulation function of MenSCs using 1000 factors included protein array and bioinformatic analysis.

## Materials and methods

### MenSCs isolation, culture and characterization

MenSCs were isolated from menstrual blood as previously described [11]. Briefly, menstrual blood was collected from females aged 25–40 years old using menstrual cups. Mononuclear cells in the menstrual blood were isolated using Ficoll (cat NO., LTS1077, TBD, Tianjin, China). Then, the mononuclear cells were re-suspended by DMEM completed medium (cat NO., ZQ-101, Zhong Qiao Xin Zhou Biotech, Shanghai, China) and cultured in a 37 °C, 5% CO_2_ saturation incubator. After 24 h of incubation, unattached cells were removed and fresh medium was added. Once cells reached 80% confluence, cells were sub-cultured as a ratio of 1:2 or 1:3. According to the minimal criteria for defining MSCs given by International Society for Cellular Therapy [20], the phenotype of passage 3 MenSCs was assessed by fluorescence-activated cell sorting (FACS) using MSCs surface markers detection kit (cat NO.562245, BD Pharmingen, San Jose, United States). Besides, the expression of CD146 (65181-1-lg, Proteintech, Wuhan, China) was also detected by flow cytometry.

Mouse IgG1 isotype control (66360-1-Ig) was used as control group. Alexa 488 conjugated goat anti-mouse secondary antibody (A28175, Invitrogen) was used to stain MenSCs. Meanwhile, the differentiation ability of passage 3 MenSCs was assessed by adipogenesis, osteogenesis, and chondrogenesis using differentiation induction kits (cat NOs., CHEM-200010, CHEM-200011, CHEM-200012, Unicorn League Biotech, Shanghai, China).

### The culture of BV2 and HAPI microglia cells

BV2 cell line was purchased from China Center for Type Culture Collection (CCTCC, Wuhan, China) and grown in DMEM completed medium (cat NO., ZQ-101). HAPI cell line was supplied by Prof. Wenqiang Li (the First Affiliated Hospital of Xinxiang Medical University), and it was used between passage 7 and 15 to avoid any phenotypic drift. HAPI cell line was maintained in DMEM high glucose medium (cat NO., 10-013-CVRC, Corning) supplemented with 10% FBS (cat NO., 164210-50, Procell, Wuhan, China) and 100 U/ml of penicillin–streptomycin. Cells were maintained at 37°C in a humidified incubator under a 95% air/5% CO_2_ condition.

### 6-OHDA treatment and MenSCs co‑culture

BV2 and HAPI cells were seeded on 14 mm glass cover slips at a density of 6 × 10^4^ cells per cover slip. After reaching 70% confluence, BV2 and HAPI cells were exposed to 300 μM and 100 μM 6-OHDA (Cat NO. H4381, Sigma-Aldrich, St. Louis, MO), respectively. Spontaneously, 6-OHDA exposed cells were in-directly co-cultured with MenSCs (6-OHDA + MenSCs group) or only medium (6-OHDA + DMEM group) with a transwell system (662641, Greiner Bio-One) for 24 h. Next, the morphology of BV2 and HAPI cells was analyzed with phase contrast and immunofluorescence. The RNA and protein of cells from different groups were isolated for following studies.

### Cell viability assay

70% confluent BV2 and HAPI cell lines in 96-well plate were treated with different concentrations of 6-OHDA for 24 h. Then, cell viability was assessed by PrestoBlue reagent (Invitrogen, USA) according to the manufacturer’s protocol. Fluorescence was read at Ex544 nm/Em590 nm using a microplate reader (BMG Labtech, Offenburg, Germany). The cell viability was calculated according to the following formula: Cell viability (%) = [(fluorescence of treatment group − blank/fluorescence of control group − blank)] × 100%.

### Immunocytofluorescence

For immunocytofluorescence, BV2 or HAPI cells were fixed with 4% PFA, permeabilized with 0.1% Triton X-100, and blocked with blocking buffer (P0260, Beyotime, Shanghai, China) for 1 h at room temperature. Afterwards, primary antibody against Iba-1 (153696, Abcam, 1:500) was incubated overnight at 4℃, followed by Cy3-conjugated goat anti-rabbit secondary antibody (A10520, Invitrogen, 1:500) for 2 h at room temperature. Nuclei were counterstained with DAPI. Images (× 200 magnification) were photographed using an inverted microscope (Leica, DMI3000B, Germany). Cells were analyzed in five randomly selected fields per cover-slip, with three cover-slips for each group, in three independent sets of experiments. ImageJ software was used to measure the length of process. If the length of the process is at least twice longer than the diameter of the cell body, the cell is considered being activated.

### Total RNA extraction and quantitative RT-PCR

Rats were euthanized by intraperitoneal injection of 250 mg/kg pentobarbital sodium and the right side of rat brain was isolated. Total RNA of BV2 cells, HAPI cells or brain tissues from different groups was extracted using TRIzol reagent (DP424, Tiangen) according to the manufacturer’s instruction. For tissue RNA extraction, the right side of rat brain was lysed by tissue lyser first and other steps were the same with cell RNA extraction. The concentration and quality of RNA was determined by a Nanodrop2000 spectrophotometer (Thermo Fisher Scientific Co., Ltd, Waltham, MA, USA). The reverse transcription reaction with 1 μg of total RNA was carried out with a cDNA synthesis kit (KR116, Tiangen Biotech Co., LTD, Beijing, China). Thereafter, PCR amplification was performed using SYBR Green PCR Master Mix (208054, Qiagen) and Roche LightCycler® 960 system under the following condition: 95℃ for 2 min, 40 cycles of denaturing at 95 ℃ for 5 s and combined annealing/extension at 60°C for 10 s, melting curve analysis and holding at 4℃. The critical threshold (ΔCT) value of interest genes was normalized to the housekeeping gene glyceraldehyde 3-phosphate dehydrogenase (GAPDH). The results were calculated using 2^−ΔΔCT^ method. The primers of qRT-PCR are listed in Additional file 1: Table S1.

### Animals and experimental design

Adult male SD rats (180–200 g, 42–48 days old) were purchased from Beijing Vital River Laboratory Animal Technology Co., Ltd. (Beijing, China; License No. SCXK (Jing) 2016-0011). Animals were maintained under standard laboratory conditions with 12 h light/dark cycle, 23 ± 2℃, and 45–60% humidity. Rats were kept in plexiglass cages (max: 2 rats/cage) and were watered and fed ad libitum. Cages of animals were put randomly on the shelf without fixed order or place. Bedding was changed once a week, but without changing bedding 2 days before and after drug or cell injection. After adapting to the new environment for 5–7 days, animals were fasted for 12 h and were used to construct PD model. All protocols for the animal experiments were registered and approved by the Animal Care and Use Committee of Xinxiang Medical University. This study was carried out in compliance with the ARRIVE guidelines. All efforts were made to minimize the number of animals used and their suffering. All the experimental conditions were performed in a within-subject randomized-order design. Surgery and behavior test were done randomly without any fixed order. Rat samples including brain, serum, and CSF in different groups were analyzed randomly to remove any deviation from the drug or cell injection order. All experimental data were collected and analyzed in a blinded fashion; the operator and designer were blinded to each other.

### PD model construction

The animals were randomly divided into two groups by lottery method: Sham group (N = 18) and PD group (N = 36). Because of the inevitable mortality in the process of surgery and failure rate of PD model construction, 15 rats of sham group and 30 rats of PD group could be achieved finally. Rats exhibiting signs of humane endpoints were immediately sacrificed. Brain stereotactic injection of 6-OHDA to medial forebrain bundle (MFB) region was used to construct PD model. Succinctly, rats were anesthetized with 4% isoflurane using an anesthetic gas machine and fixed on a brain stereoscopic injection equipment. 2.5% isoflurane and 1 L/min oxygen was used for continuous anesthesia during surgery. Rats were unilaterally injected with either 4 μl 6-OHDA (2.5 μg/μl dissolved in 0.2 mg/ml ascorbic acid), or 4 μl 0.2 mg/ml ascorbic acid (dissolved in 0.9% saline) using a 10 μl Hamilton syringe with a 30-gauge flat needle (Hamilton, Bonaduz, Switzerland). The injection coordinates related to bregma was AP = − 4.36 mm, ML = − 1.7 mm, DV = − 8.3 → − 8.0 mm, according to the Paxinos and Watson brain atlas [21]. To avoid any overflow, the injection was at a slow rate of 1 μl/min and the needle was left in place for 10 min before it was pulled out.

### MenSCs transplantation

Five weeks after surgery, 30 PD rats were randomly divided into two groups by lottery method: PD + PBS (N = 15), and PD + MenSCs (N = 15). The anesthesia method was the same with PD model construction. MenSCs or PBS were transplanted into SNc (coordinates related to Bregma: AP = − 5.3 mm, ML = 1.8 mm, DV = − 7.4 mm) and four different Str sites (coordinates related to Bregma: AP = − 1.3 mm, ML = 4.7 mm, DV = − 4.5 mm and − 4.0 mm; AP = − 0.4 mm, ML = 4.3 mm, DV = − 4.5 mm and − 4.0 mm; AP = 0.4 mm, ML = − 3.1 mm, DV = − 4.5 mm and − 4.0 mm; AP = 1.3 mm, ML = 2.7 mm, DV = − 4.5 mm and − 4.0 mm) of brain [22]. In the SNc, 6-OHDA animals received once injection of either 4.0 μl MenSCs (2 × 10^5^, dissolved in PBS) or 4.0 μl PBS at a rate of 1.0 μl/min. In the striatum, 6-OHDA animals received either 2.0 μl MenSCs (5 × 10^4^, dissolved in PBS) or 2.0 μl PBS in each coordinate of striatum at a rate of 0.5 μl/min. After each injection, the needle was left in place for 8 min in the SNc, and 4 min in the Str to avoid any back flow up the needle tract.

### Open field

The locomotor activity of rats was conducted using an animal movement tracking system EthoVision XT (Noldus, Wageningen, Netherlands). Each rat was placed in the center of a black Plexiglass cage (100 × 100 × 40 cm) and allowed for free activity. Data were collected for 5 min per rat. Total distance travelled and ambulatory episodes were automatically analyzed from video recordings using EthoVision XT software.

### Rotarod test

Before the formal test, all rats were pre-trained on the rotarod for continuous 3 days. After exercise, rats were placed on an automated 6-lane rotarod apparatus (RWD, Shenzhen, China) and were tested in consecutive three trials at 15 rpm and 25 rpm for a maximum of 300 s at each speed with an interval of 20 min. An accelerating mode was used in the test, from 0 to 15 rpm or 0 rpm to 25 rpm over 300 s. This test was conducted to evaluate the motor coordination and balance of rats. The duration time of each animal that was able to stay on the rod was recorded as the latency to fall. The results were calculated as the average of three trials.

### Rotameter test

In order to select the animals that were truly lesioned upon 6-OHDA injections and assess the therapeutic effect of MenSCs, the rotameter test was performed at indicated time points. Briefly, 0.05 mg/ml apomorphine hydrochloride (Sigma, St. Louis, MO, USA) solution was freshly prepared by dissolving in 1% of ascorbic acid in 0.9% of NaCl. Animals’ necks were subcutaneously injected with 0.05 mg/kg apomorphine hydrochloride (A4393, sigma, St. Louis, MO, USA). Afterwards, the complete contralateral rotation (360°) of the rats in 30 min was counted in a mental bowl. More than 7 r/min was considered successful PD models and unsuccessful models will be excluded.

### Collection of cerebrospinal fluid (CSF) and serum

Seven weeks after MenSCs transplantation, rats were deeply anesthetized with ketamine (75 mg/kg) combined with medetomidine (0.5 mg/kg) by intraperitoneal injection. Then, cerebrospinal fluid was collected from the cisterna magna of the fourth ventricle using a capillary needle. 100–200 μl CSF was collected and stored at − 70°C for further analysis. The CSF collection protocol was referred to Liu L et al. [23]. Blood was collected from orbital venous plexus and serum was prepared by centrifuging the clotted blood at 3000 rpm for 5 min.

### Tissue processing and immunohistochemistry

After collecting the CSF, rats were transcardially perfused with 0.9% saline followed by 4% paraformaldehyde. Then, brains were dissected and post-fixed in 4% paraformaldehyde for 48 h at 4 °C. Fixed brains were dehydrated in gradient ethanol followed by xylene before embedding in paraffin wax. Serial coronary sections (5-μm) were prepared using a rotary microtome (Leica Model RM 2145, Germany). Based on the anatomical landmarks according to Paxino and Watson, for each rat, 5 slices containing striatum region (bregma 1.8 mm, 1.2 mm, 0.60 mm, 0.12 mm, − 0.60 mm), and 5 slices containing SN region (bregma − 4.56 mm, − 5.04 mm, − 5.52 mm, − 6.00 mm, − 6.48 mm) were collected for further immunohistochemistry and analysis.

Brain sections were deparaffinized in xylene and rehydrated in graded ethanol before undergoing antigen retrieval. 0.3% hydrogen peroxide dissolved in absolute methanol was used to inactivate endogenous peroxidase for 15 min. Sections were incubated with a primary antibody (rabbit anti-TH, PA585167, Invitrogen, 1:1000) at 4 °C overnight, followed by incubation with HRP-conjugated secondary antibody (A16096, Invitrogen, goat anti-rabbit, 1:5000) at RT for 1 h. Brown color was developed using diaminobenzidine staining and sections were then counterstained with hematoxylin. To circumvent arbitrary delineation, a Pannoramic MIDI system (3DHISTECH, Budapest, Hungary) was used to achieve the whole slide image. The amount of TH + fibers in the striatum region was analyzed by detecting optical density of each slice using the ImageJ software version 1.51 (National Institutes of Health, U.S.A.). The number of TH + cells in the SN and VTA regions were counted using ImageJ software. Results were expressed as a percentage of the contralateral side on the same section.

### Western blot

Seven weeks after MenSCs transplantation, rats were euthanized by intraperitoneal injection of 250 mg/kg pentobarbital sodium and brain was quickly detached on ice. RIPA buffer supplemented with protease inhibitor cocktail was used to extract protein from brain tissue. After denature, 20 μg of total protein was fractionated by SDS-PAGE. Nonspecific binding was blocked with 5% w/v BSA (A1933, Sigma) in Tris-buffered saline-Tween containing 0.1% Tween-20 (TBST) for 1 h. Primary antibodies, unless specified, were purchased from Proteintech, including rabbit anti-iNOS (18985-1-AP, 1:500), rabbit anti-TH (PA585167, Invitrogen, 1:1000), mouse anti-Arg1 (66129-1-Ig, 1:5000), and mouse anti-β-actin (66009-1-Ig, 1:10000) as the internal control. Primary antibodies were incubated overnight at 4 °C followed by HRP-conjugated secondary antibodies (A16096, Invitrogen, goat anti-rabbit, 1:10000 or 62-6520, Invitrogen, goat anti-mouse, 1:5000) for 2 h at room temperature. Immunoblotted protein signals were visualized with ECL enhanced chemiluminescent substrate kit (WBKLS0050, Millipore) following manufacturer’s instructions. Densitometry of the western blot protein bands was analyzed using Image J software v5.2.1. β-actin was used as an internal control for western blot analysis.

### Protein microarray analysis

Protein microarray kits (QAR-INF-1-2, RayBiotech Life Inc., Norcross, GA, USA) were used to detect 10 kinds of inflammatory factors in the CSF and serum (N = 5 per group), including IFN-γ, IL-1α, IL-1β, IL-2, IL-4, IL-6, IL-10, IL-13, MCP-1, and TNF-α.

Another protein array kit (GSH-CAA-X00-1, RayBiotech Life Inc., Norcross, GA, USA) was used to detect the amount of 1000 kinds of factors in the conditioned medium of MenSCs (MenSCs-CM). The preparation method of MenSCs-CM referred to our previously published paper [11]. Signals were scanned by InnoScan 300 Microarray Scanner (Innopsys, Carbonne, France). The measured fluorescence intensities were normalized to that of the internal positive controls.

### Bioinformatics analysis

Gene Ontology and Kyoto Encyclopedia of Genes and Genomes (KEGG) pathway analysis were performed using DAVID Bioinformatics Resources v2022q2 (https://david.ncifcrf.gov).

### Statistical analysis

Graphs generation and statistical analysis were performed using Prism 8.0.2 software (GraphPad, San Diego, CA, USA). Data were expressed as means ± standard deviation (SD). With the mRNA expression of M1 and M2 markers as primary outcomes, if the true difference in the PD + MenSCs and PD + PBS means is 0.5, we will need to study 5 subjects in each group to reject the null hypothesis that the population means of each group are equal with probability (power) 0.9. The Type I error probability associated with this test of this null hypothesis is 0.05[24]. Therefore, our samples for animal work are 5 for each group. Data normality was detected by Shapiro–Wilk test. Homogeneity of variance was detected by Brown-Forsythe test. For data that fit a normal distribution and homogeneity of variance, one-way ANOVA followed by Tukey's post hoc multiple comparison was used for comparison among 3 groups. For data that does not conform to a normal distribution, nonparametric Kruskal–Wallis test was used for comparison among 3 groups. Values of *p* < 0.05 were considered statistically significant.

## Results

### Characterization of MenSCs

**Fig. 1:**
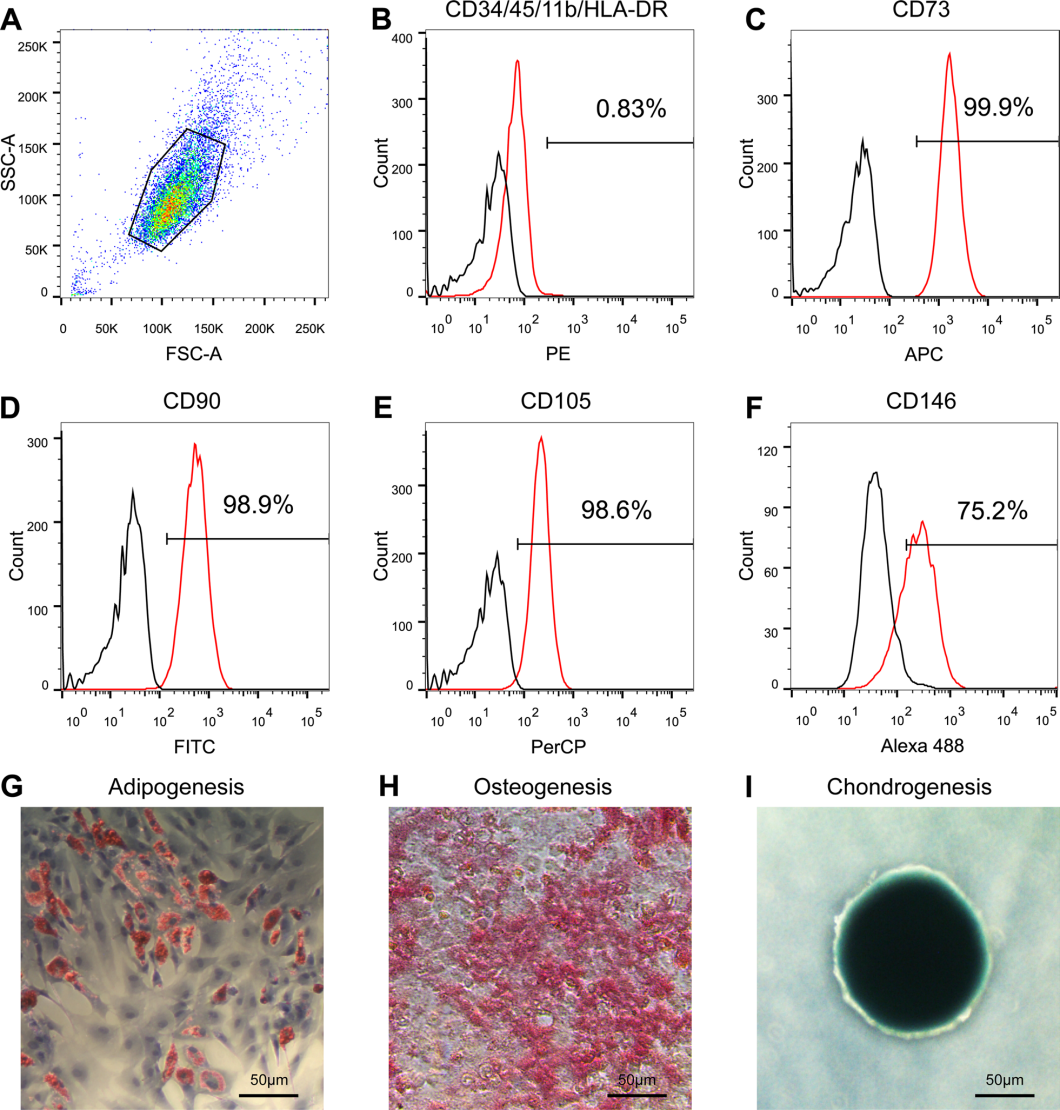
Characterization of MenSCs by immunophenotyping and mesodermal differentiation assay. **A** Cell population for further analysis was selected. **B**–**F** The expression of MenSCs surface markers was detected by flow cytometry. Red lines and blank lines represented for surface markers and relative isotype control, respectively

Figure 1A showed the gated population for further analysis. Figure 1B showed MenSCs were negative for CD34, CD45, CD11b, and HLA-DR, and the total positive cells were less than 2% as measured by flow cytometry. Figure 1C–E showed MSCs were positive for CD73-APC, CD90-FITC, and CD105-PerCP, and positive cells were more than 98% for each marker, which meet the minimum criteria of MSCs reported by International Society for Cellular Therapy (ISCT). Figure 1F showed 75.2% MenSCs were positive for CD146. Figure 1G–I showed MenSCs had capacities to differentiate to osteoblasts, adipocytes and chondroblasts under differentiation induction medium.

### MenSCs suppressed 6-OHDA-induced microglia activation and inflammation in vitro

**Fig. 2:**
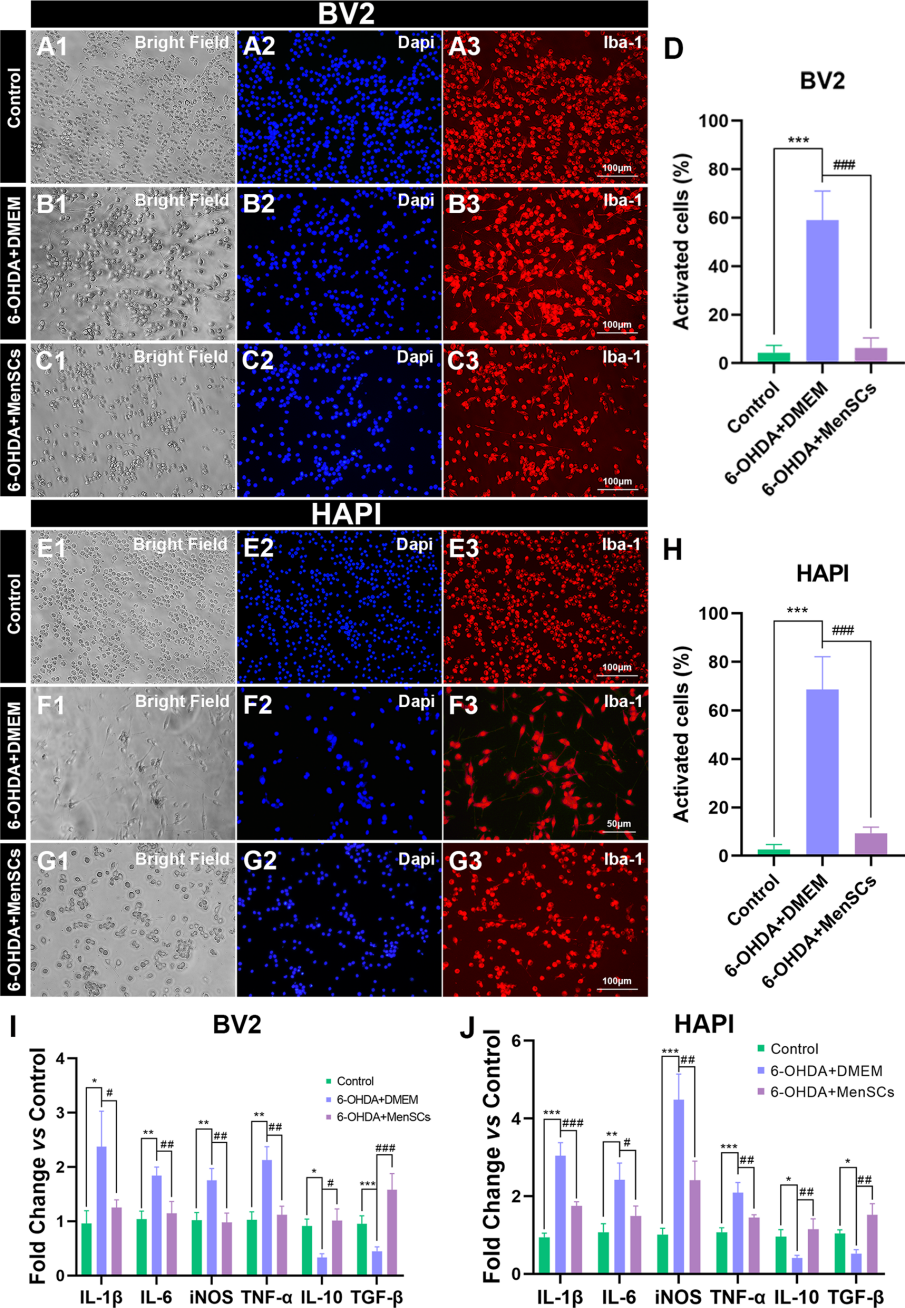
MenSCs decreased the number of activated microglia cells and attenuated 6-OHDA induced inflammation in vitro. Iba-1 was used to mark BV2 (**A1**–**C3**) and HAPI cells (**E1**–**G3**) using immunofluorescence. **A1**–**G1** The morphology of BV2 and HAPI cells was observed and captured under bright field. **A2**–**G2** Cell nucleus were stained with DAPI. **A3**–**G3** The expression of Iba-1. **D**, **H** the percentage of activated BV2 cells (**D**) and HAPI cells (**H**). **I**, **J** Pro-inflammatory and anti-inflammatory factors in BV2 and HAPI cells were detected by qRT-PCR. One-way ANOVA followed by Tukey’s post hoc test was used for statistical analysis. ^ns^*p* > 0.05, ^*^p < 0.05, ^**^p < 0.01, ^***^p < 0.001, ^#^*p* < 0.05, ^##^*p* < 0.01, and ^###^*p* < 0.001

The dose–effect curve of 6-OHDA for BV2 and HAPI cells is shown in Additional file 2: Fig. S1. BV2 and HAPI cell lines were exposed to 6-OHDA and indirectly co-culture with MenSCs (6-OHDA + MenSCs) or DMEM medium (MenSCs + DMEM) for 24 h. Then, the morphology of BV2 and HAPI was observed under bright field and Iba-1 was used to label microglia cells. As shown in Fig. 2A1–A3 and E1–E3, the normal BV2 and HAPI cells are generally round shape. 6-OHDA treatment obviously induced BV2 (Fig. 2B1–B3, D, ^**^p < 0.01) and HAPI (Fig. 2F1–F3, H, ^***^p < 0.001) cells extending 1–2 finely delineated processes and elicited microglia activation. The application of MenSCs significantly attenuated 6-OHDA induced microglia activation in BV2 (Fig. 2C1–C3, D, ^##^*p* < 0.01) and HAPI cells (Fig. 2G1–G3, H, ^###^*p* < 0.001). Consistently, 6-OHDA treatment significantly increased the mRNA expression of pro-inflammatory factors including *IL-1β*, *IL-6*, *iNOS*, *TNF-α* and decreased the mRNA expression of anti-inflammatory factors including *IL-10* and *TGF-β* in BV2 cell line (Fig. 2I, ^*^p < 0.05, ^**^p < 0.01, ^***^p < 0.001). Similar results were achieved in HAPI cell line except for the *TGF-β* (Fig. 2J, ^ns^*p* > 0.05, ^*^p < 0.05, ^***^p < 0.001). Furthermore, MenSCs inhibited inflammation by down-regulation *IL-1β*, *IL-6*, *iNOS*, *TNF-α and* up-regulation *IL-10* and *TGF-β* (Fig. 2I, J, ^#^*p* < 0.05, ^##^*p* < 0.01, ^###^*p* < 0.001).

### MenSCs improved the motor function of PD rats

**Fig. 3:**
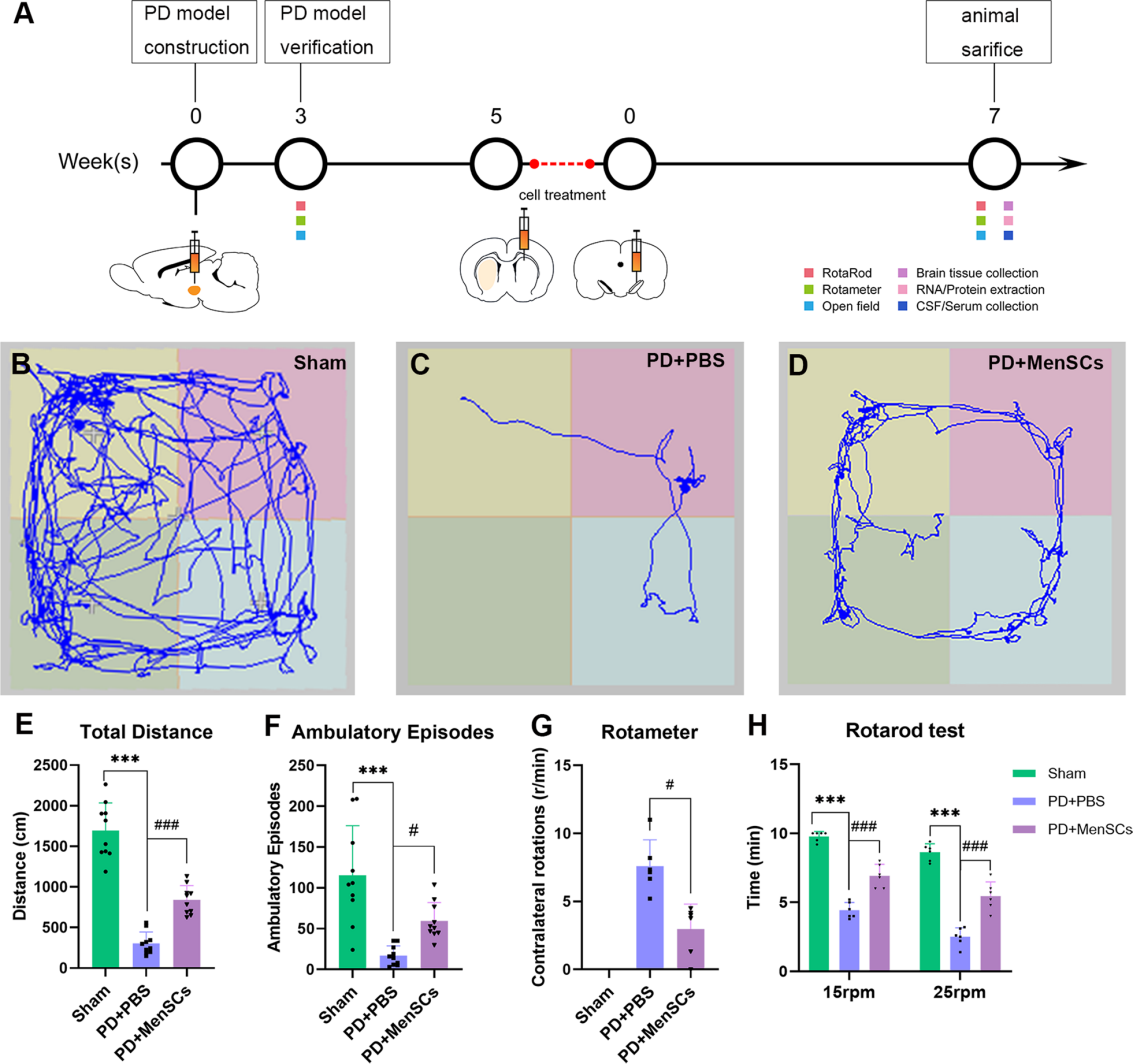
Experimental design and animal behavior test. **A** Schematic summary of the experimental procedures. **B**–**D** Open field test showed the movement path of rats. **E**, **F** Statistical analysis of movement distance and ambulatory episodes of rats. **G** Rotameter results showed contralateral rotations of rats. **H** Rotarod test results showed the duration time of rats staying on the rod. One-way ANOVA followed by Tukey’s post hoc test was used for statistical analysis. ^***^p < 0.001, ^#^*p* < 0.05, and ^###^*p* < 0.001

In order to explore the impact of MenSCs transplants on the motor performance of the PD rats, behavioral assessment including open field (n = 10 per group), rotameter (n = 5 per group), and rotarod tests (n = 5 per group) were performed at 7 weeks after transplantation (Fig. 3). Figure 3A shows schematic summary of the experimental procedures. The open field results showed that the rats of PD + PBS group had significantly less movement and ambulatory episodes than the rats of sham group (Fig. 3B, C, E, F, ^***^p < 0.001). The transplantation of MenSCs improved the motor function of PD rats compared to PBS treatment group (Fig. 3C–F, ^#^*p* < 0.05, ^###^*p* < 0.001). Besides, rotameter test results (Fig. 3G) showed the rats of PD + PBS group demonstrated 7.59 ± 1.94 r/min contralateral rotation behavior after injection with apomorphine (^***^p < 0.001 *vs* sham group). We observed an obvious reduction of contralateral rotation after MenSCs treatment (^###^*p* < 0.001 *vs* PD + PBS group). Regarding balance and motor coordination, rotarod test (Fig. 3H) showed PBS treatment PD rats could stay on the rod for 4.42 min ± 0.58 min and 2.5 min ± 0.66 min at 15 rpm and 25 rpm, respectively, which was significantly less than the sham group (9.78 min ± 0.35 min at 15 rpm and 8.63 ± 0.61 min at 25 rpm, ^***^p < 0.001). MenSCs treatment significantly elongated the time of latency to fall to 6.90 min ± 0.93 min at 15 rpm and 5.50 ± 1.15 min at 25 rpm (^###^*p* < 0.001).

### MenSCs restored TH deficits in the brain of PD rats

**Fig. 4:**
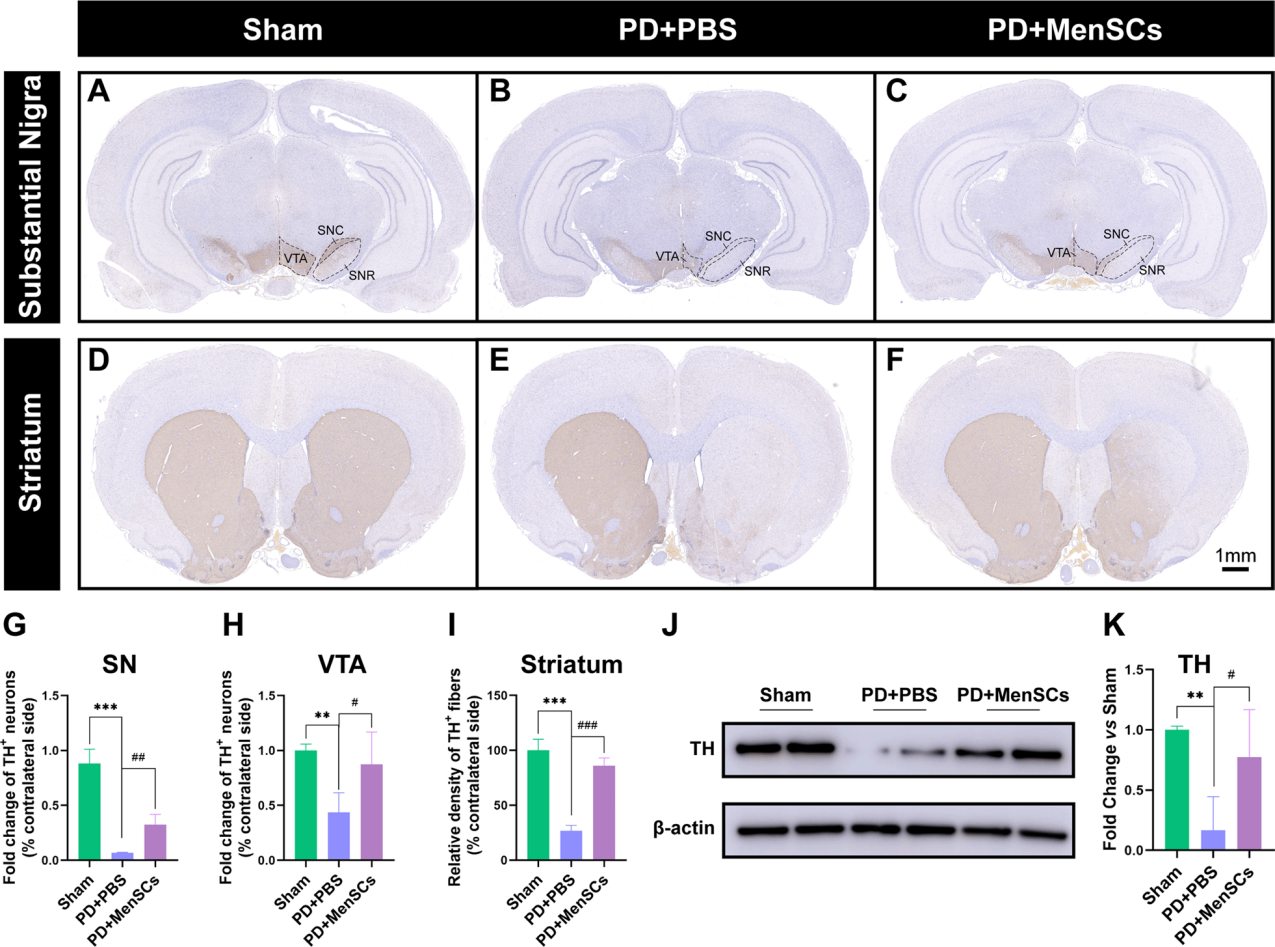
The expression of TH in the SN, VTA and striatum regions. **A**–**C** The expression of TH in the VTA and SN regions was detected by immunohistochemistry. **D**–**F** TH positive fibers in the striatum region was detected by immunohistochemistry. **G**–**I** statistical analysis of TH positive neurons and fibres.** G** and** H** showed the fold change of TH positive neurons in right SN/VTA regions compared to that in contralateral side. Image J software was used to count the number of TH positive neurons in the SN and VTA regions. **I** showed the relative density of TH positive fibers in right striatum regions compared to that in contralateral side. **J** The expression level of TH in brain was detected by WB. Full-length blots were presented in Additional file 3: Fig. S2 D, E.** K** Statistical analysis of WB results. Abbreviations: SN, substantia nigra; TH, tyrosine hydroxylase; VTA, ventral tegmental area. One-way ANOVA followed by Tukey’s post hoc test was used for statistical analysis. ^**^p < 0.01, ^***^p < 0.001, ^#^*p* < 0.05, ^##^*p* < 0.01, and ^###^*p* < 0.001

To address the neuroprotective function of MenSCs on PD rats, histological and WB analysis for the expression of TH was performed (n = 5 per group). As shown in Fig. 4, in the sham group, the TH expression in the SN, ventral tegmental area (VTA), and striatum regions was similar between the left and right sham lesioned section (Fig. 4A, D). 6-OHDA injection significantly induced the loss of dopaminergic neurons in the SN and VTA regions, and decreased TH-positive fibers in the striatum compared to the sham group (Fig. 4B, E, G-3I, ^**^p < 0.01, ^***^p < 0.001). After MenSCs treatment, the number of TH-positive neurons increased compared to the PD + PBS group (^#^*p* < 0.05, ^##^*p* < 0.01). Similar results were also observed in the striatum region (Fig. 4D–F, I). Consistently, WB results showed higher expression of TH proteins in the PD + MenSCs group compared to PD + PBS group (Fig. 4J, K, ^#^*p* < 0.05). TH belt was cropped between 35 kDa-75 kDa. β-actin belt was cropped between 35 kDa-65 kDa. These results suggested that MenSCs may play a role in promoting dopaminergic neurons survival in PD rats.

### MenSCs regulated the expression of inflammatory factors in the CSF and serum of rats

**Fig. 5:**
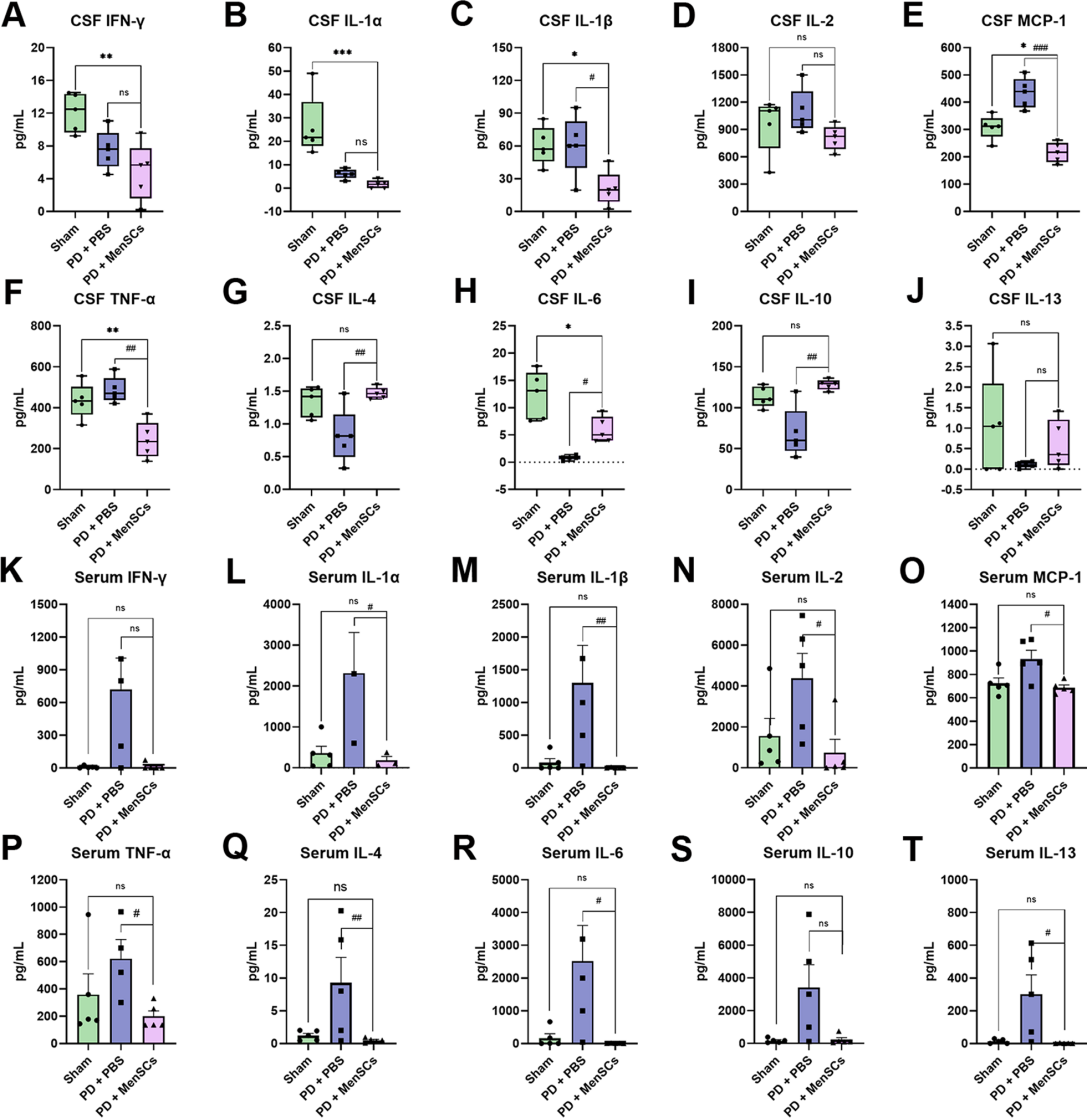
Inflammatory factors detection by protein array. **A**–**J** The amount of pro-inflammatory and anti-inflammatory factors in the CSF.** K**–**T** The amount of pro-inflammatory and anti-inflammatory factors in the serum. One-way ANOVA followed by Tukey’s post hoc test was used for statistical analysis. ^ns^*p* > 0.05, ^*^p < 0.05, ^**^p < 0.01, ^***^p < 0.001, ^#^*p* < 0.05, ^##^*p* < 0.01, and ^###^*p* < 0.001

As shown in Fig. 5, compared with PD + PBS group, the transplantation of MenSCs significantly down-regulated the amount of pro-inflammatory factors IL-1β (Fig. 5C, ^#^*p* < 0.05), MCP-1 (Fig. 5E, ^###^*p* < 0.001), and TNF-α (Fig. 5F, ^##^*p* < 0.01) in the cerebrospinal fluid (CSF). Although there was no significant statistical difference (^ns^*p* > 0.05), the amount of pro-inflammatory factors IFN-γ (Fig. 5A, 4.86 ± 3.49 pg/mL), IL-1α (Fig. 5B, 1.61 ± 1.76 pg/mL), and IL-2 (Fig. 5D, 810.12 ± 134.60 pg/mL) in PD + MenSCs group was less than those in PD + PBS group (7.55 ± 2.39 pg/mL for IFN-γ, 6.05 ± 2.03 pg/mL for IL-1α, 1095.92 ± 245.76 pg/mL for IL-2). In addition, the transplantation of MenSCs significantly up-regulated the level of anti-inflammatory factors IL-4 (Fig. 5G, ^##^*p* < 0.01), IL-6 (Fig. 5H, ^#^*p* < 0.05), and IL-10 (Fig. 5I, ^##^*p* < 0.01) compared with PD + PBS group. The level of IL-13 (Fig. 5J, 0.59 ± 0.59 pg/mL) in the CSF of PD + MenSCs group was also higher than that in PD + PBS (0.11 ± 0.07 pg/mL) group without statistical difference (^ns^*p* > 0.05). After MenSCs treatment, the level of IL-2, IL-4, IL-10, and IL-13 restore to be similar to that of the sham group (Fig. 5D, G, I, J, ^ns^*p* > 0.05). Interestingly, the level of IFN-γ, IL-1α, IL-1β, MCP-1, and TNF-α in the CSF of PD + MenSCs group is even lower than that of sham group (Fig. 5A–C, E–F, ^*^p < 0.01, ^**^p < 0.01).

Compared with PD + PBS group, the amount of pro-inflammatory factors IL-1α, IL-1β, IL-2, MCP-1, and TNF-α in the serum of PD + MenSCs group significantly decreased after MenSCs transplantation (Fig. 5L–P, ^#^*p* < 0.05, ^##^*p* < 0.01). The level of IFN-γ (Fig. 5K, 14.92 ± 31.90) in the serum of PD + MenSCs group also decreased but without significant difference compared with that of PD + PBS group (719.61 ± 641.41). Contrary to the results in CSF, the level of anti-inflammatory factors IL-4, IL-6, and IL-13 in the serum of PD + MenSCs group was significantly lower than that of PD + PBS group (Fig. 5Q–R, T, ^#^*p* < 0.05, ^##^*p* < 0.01). After MenSCs treatment, there was no statistical difference in the expression level of all the forementioned factors between PD + MenSCs group and sham group (Fig. 5K–T, ^ns^*p* > 0.05), which indicated that MenSCs could facilitate the inflammatory factors to restore to normal level.

### MenSCs could modulate M1/M2 polarization

**Fig. 6:**
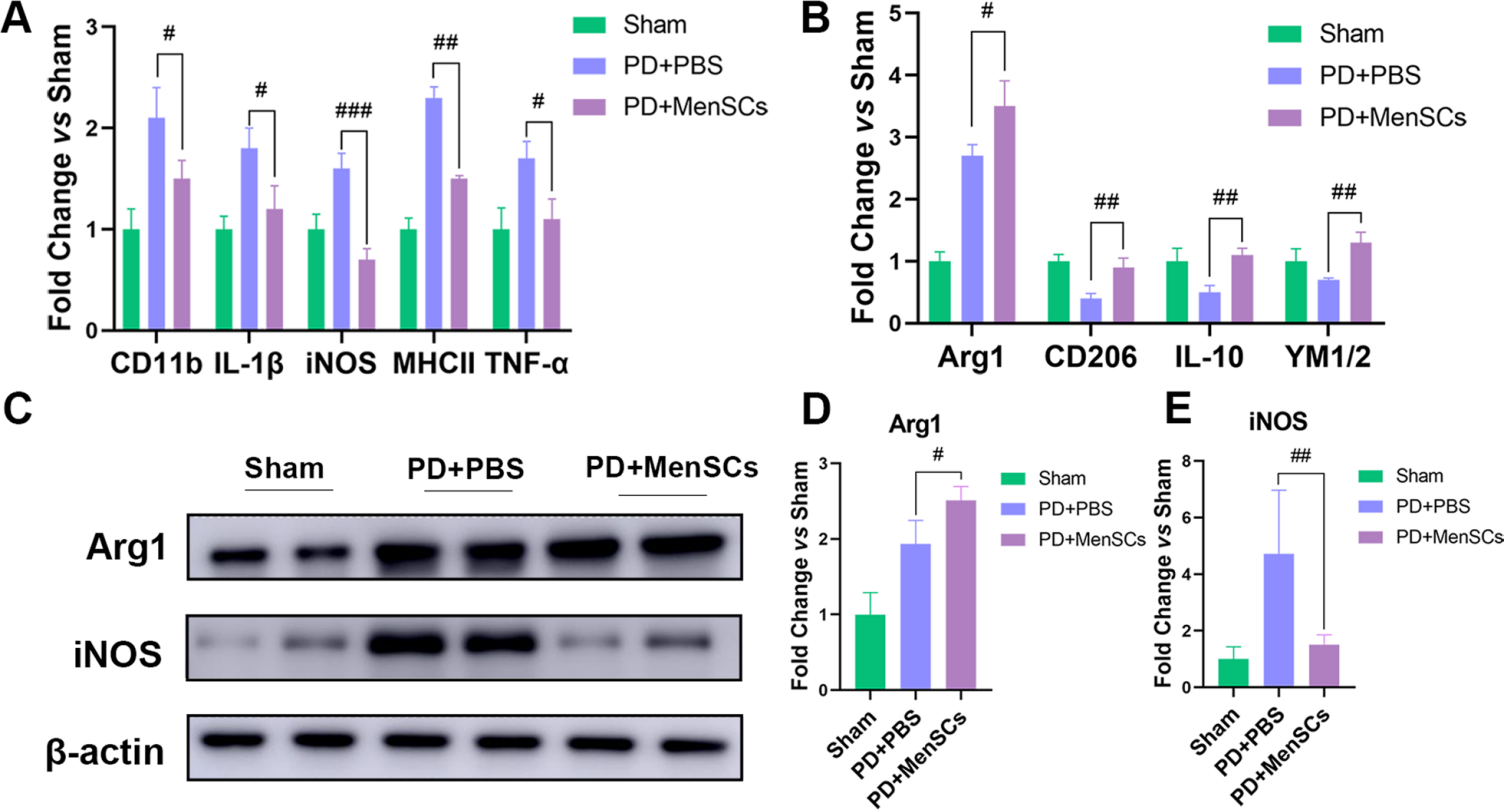
The expression of M1 and M2 microglial markers in rat brain detected by qRT-PCR and WB. **A** qRT-PCR was used to detect markers of M1 phenotype microglia including *CD11b*, *IL-1β*, *iNOS*, *MHCII* and *TNF-α*. **B** qRT-PCR was used to detect markers of M2 phenotype microglia including *Arg1*, *CD206*, *IL-10*, and *YM1/2*. **C** WB results showed the represent cropped images of Arg1, iNOS, and internal control β-actin. Full-length blots were presented in Additional file 3: Fig. S2 A-C. **D** Statistical analysis of the WB results of Arg1 and iNOS. One-way ANOVA followed by Tukey’s post hoc test was used for statistical analysis. ^#^*p* < 0.05, ^##^*p* < 0.01, and ^###^*p* < 0.001

After MenSCs were transplanted into the brain of PD rats, to evaluate the effect of transplanted MenSCs on two phenotypes of activated microglia cells in vivo, the RNA and protein of the right side brain were extracted (n = 5 per group). Then, the mRNA expression levels of the markers of M1/M2 phenotype microglia were determined by qRT-PCR. Results showed that MenSCs significantly down-regulated the expression of M1 microglia markers *CD11b*, *IL-1β*, *iNOS*, *MHCII,* and *TNF-α* and meanwhile up-regulated the expression of M2 microglia markers *Arg1*, *CD206*, *IL-10*, and *YM1/2* (Fig. 6A, B, ^#^*p* < 0.05, ^##^*p* < 0.01, ^###^*p* < 0.001 *vs* PD + PBS group). In addition, WB was performed to detect the protein expression levels of M1 marker iNOS and M2 marker Arg1. Figure 6C, D represents cropped sections of Arg-1, iNOS and β-actin. Arg1 belt was cropped between 25 and 45 kDa. iNOS belt was cropped between 75 and 135 kDa. β-actin belt was cropped between 35 and 45 kDa. MenSCs treatment group had higher expression level of Arg1 and lower expression level of iNOS compared with PBS treatment group (^#^*p* < 0.05, ^##^*p* < 0.01), which was consistent with the qRT-PCR results.

### MenSCs secreted multiple molecules playing neuroregulatory roles

**Fig. 7:**
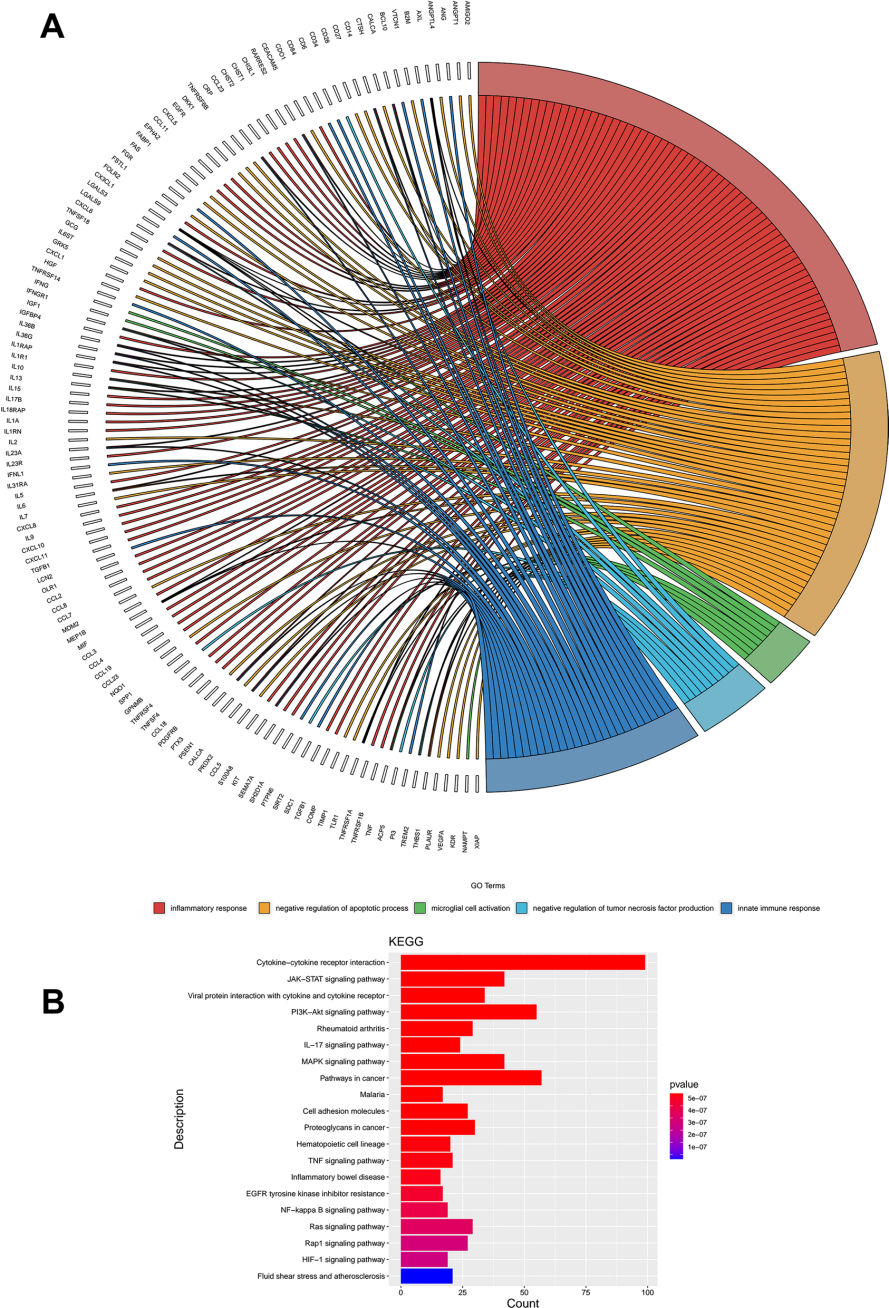
GO and KEGG enrichment analysis of proteins in the MenSCs-CM. **A** GO_Biological Process analysis. The chord diagram shows the five most noteworthy terms. **B** KEGG pathway analysis. The color of the column represents statistical significance, and the length of the column represents the number of genes enriched into the function

In order to reveal the therapeutic mechanism of MenSCs on PD, protein microarray containing 1000 factors was used to detect protein components in the conditioned media of MenSCs. 445 proteins with fluorescence intensity greater than 1000 were selected for further Gene Ontology Biological Process (GO-BP) and Kyoto Encyclopedia of Genes and Genomes (KEGG) pathway analysis using DAVID Bioinformatics Resources. 176 biological process and 58 pathways were enriched and the results were listed in Additional file 1: Tables S2 and S3. Among the 176 biological processes, 5 biological processes of interest were selected to draw chord diagrams, including inflammatory response, negative regulation of apoptotic process, microglial cell activation, negative regulation of TNF-α production, and innate immune response (Fig. 7A). These biological processes may partly explain the therapeutic functions of MenSCs on promoting the survival of DAergic neurons and anti-inflammation by regulating M1/M2 polarization. The top 20 KEGG pathways were demonstrated in Fig. 7B.

## Discussion

6-OHDA unilateral lesioned rats showed significant ~ 93% dopaminergic (DAergic) neurons loss in SN region, i.e., brain A8 and A9 areas. Although a lesser extent, VTA region (i.e., A10 area) was also affected by 6-OHDA showing ~ 57% TH^+^ neuronal loss. A10 area is the origin of the mesocorticolimbic DAergic projections to nucleus accumbens and prefrontal cortex, and are mainly involved in reward, motivation and hedonia. The abnormality of mesocorticolimbic DAergic network will contribute to non-motor features, such as depression, anhedonia, anxiety, apathy, etc. [25, 26]. PD patients suffer from both motor and non-motor symptoms, such as depression, anxiety, sleep disorders, cognitive impairment, and pain, etc., which contribute to severe disability, impaired quality of life, and shortened life expectancy [25]. Miguel et al. demonstrated that 6-OHDA unilateral injected rats showed depressive-like behavior detected by sucrose preference test [26]. Although we mainly focused on the motor function evaluation of PD rats, the locomotor activity in open field test reflected not only motor ability but also motivation to explore new environment [27]. When rats entered a strange open field, they preferred to move in the periphery area instead of the central area because of the fear of the new environment and their thigmotaxis habitual nature. However, the exploration characteristics of animals also motivated them to move in the central area, leading to conflict behaviors [27]. It has been reported that depressed animals showed lesser conflict behaviors compared to healthy animals [27]. According to Fig. 3B–D, PBS treated rats were unactive and their moving was only limited to periphery area, while MenSCs treated rats had stronger motivation to explore the new area. This result suggest MenSCs may have the ability to improve the depression of PD rats. More experiments need to be designed to further assess the non-motor behaviors of PD rats, such as anhedonic, anxiety and mood, and cognitive performance, etc., and to evaluate the therapeutic potential of MenSCs on these non-motor function impairments.

Accumulating literatures have reported that MSCs have the potential to relieve the pathology of PD [28, 29]. However, it is still controversial whether grafted MSCs could differentiate into functional DAergic neurons and integrate with endogenous remained neurons [30]. Besides, if differentiation happened, the differentiation rate was generally very low [31, 32]. Wolff et al. reported that 4 × 10^6^ endometrial stem cell allografts were transplanted into the striatum of PD monkeys, and about 0.01% of the cells could differentiate into DAergic neurons [31]. In this present study, we did not observe the neural differentiation of MenSCs in vivo (data not shown). MenSCs may play the therapeutic effect mainly by the secretion of an array of bioactive molecules and extracellular vesicles.

Previous studies have shown that MSCs derived from umbilical cord and adipose could secrete a variety of growth factors, immunomodulatory factors and angiogenic factors when transplanted into rat brain of PD, which facilitated the improvement of animals’ motor function [33, 34]. In our previous study, the conditioned medium (i.e., secretome) of MenSCs (MenSCs-CM) was co-cultured with an in vitro PD model [11]. Results demonstrated MenSCs-CM could attenuate MPP^+^-induced cytotoxicity by reducing apoptosis, inflammation, oxidative stress and mitochondrial damage [11]. Furthermore, the exosomes of MenSCs could also improve cell viability, but the degree of improvement was not as good as MenSCs-CM [11]. These results indicated the soluble factors secreted by MenSCs may play an important role in anti-apoptosis and anti-inflammation. Further evidence showed twelve kinds of neurotrophic factors were present in the MenSCs-CM, including neurotrophin family members (BDNF, β-NGF, NT-3, and NT-4/5), GDNF family of ligands members (ARTN, GDNF, NTN, PSPN), novel dopaminergic neurotrophic factor family members (CDNF, MANF), HGF and IGF-1 [11]. To further clarify the mechanisms of MenSCs in M1/M2 modulation, anti-inflammation and neuroprotection, protein array including 1000 factors was performed. Except for the above twelve kinds of neurotrophic factors, more neurotropic factors and cytokines were found in the MenSCs-CM (Additional file 1: Table S2). GO-BP analysis showed these factors involved in inflammatory response (64 factors), immune response (57 factors), negative regulation of apoptotic process (40 factors), microglial cell activation (8 factors), negative regulation of tumor necrosis factor production (10 factors), and innate immune response (26 factors) etc. These results provide a reasonable explanation for the role of MenSCs in regulating microglia, inflammation and apoptosis in this present study. Among these factors, several attracted our attention including growth differentiation factor 15 (GDF-15), angiopoietin-1 (Ang-1), thrombospondin-1/2/5, and X-linked inhibitor of apoptosis protein (XIAP).

GDF-15 is a member of the TGF-β superfamily [35]. It has been reported to play an important role in regulating apoptosis, inflammation, angiogenesis, and lipid metabolism, etc. in various debilitating conditions, including PD, Alzheimer’s disease, Huntington’s disease, and stroke [36, 37]. Exogenous GDF-15 could rescue 6-OHDA-induced apoptosis of DAergic neurons both in vitro and in vivo [35]. The function of endogenous GDF-15 was also investigated using *Gdf-15*^+/+^ and *Gdf-15*^−/−^ mice [38]. Results showed stereotactic injection of 6-OHDA significantly led to the loss of DAergic neurons in both wild and *Gdf-15* knockout mice. However, the extent of dopaminergic neuron loss in *Gdf-15* deficient mice was much higher than that in wild-type mice. Besides, the total number of microglia cells and the number of activated microglia cells were significantly lower than those of wild-type mice [38]. Although it is unknown whether the overexpression of GDF-15 in MenSCs could improve the therapeutic efficiency on PD models, BMSCs-exosomes containing GDF-15 has been reported to alleviate Aβ42-induced SH-SY5Y cell damage by suppressing cell apoptosis and inflammation [39].

Ang-1 is a well-known endothelial growth factor and serve an important role in anti-inflammation and anti-apoptosis [40, 41]. In a lipopolysaccharide-induced acute lung injury (ALI) rat model, the injection of Ang-1 modified human umbilical cord derived MSCs (hUC-MSCs) could decrease the expression of pro-inflammatory cytokines (e.g., TNF-α, TGF-β1) and increase the expression of anti-inflammatory cytokine IL-10[42]. Boris et al. reported Ang-1 treatment could inhibit primary neural apoptosis in an etoposide-induced neuronal injury model by regulating Bcl-2 family members and caspase-dependent and caspase-independent pathways [41]. Another in vitro study showed Ang-1 protect oxygen–glucose deprivation exposed rat primary neurons from death by inhibiting autophagy [43]. Venkat et al. showed Ang-1 mimetic peptide could promote neurological recovery and decrease infarct volume in type one diabetic (T1DM) rats subjected to ischemic stroke [44].

Thrombospondin-1 was initially identified as an astrocyte secretory factor involved in excitatory synapse formation [45]. Reduced thrombospondin-1 levels have been reported in Down syndrome and sporadic AD brains [45]. Thrombospondin-1 may counter inflammatory processes in multiple sclerosis [46], participate in the proliferation and differentiation of neural progenitor cells [47], mediate axon regeneration [48] and contribute to formation and modulation of dendritic spines [45]. Thrombospondin-2 (encoded by *THBS2* gene) was critical to repair blood brain barrier. *THBS2* knockout astrocytes could not recover the barrier function of mouse brain microvascular endothelial cells in vitro [49]. *THBS2* overexpression can effectively inhibit LPS-induced acute respiratory distress syndrome in vivo and promote macrophage polarization to M2 phenotype in vitro [50]. Thrombospondin-5, also called cartilage oligomeric matrix protein (COMP), was demonstrated to promote angiogenesis and protect against endothelial cell apoptosis [51].

XIAP is a key member of inhibitor of apoptosis proteins (IAPs) family. It can block apoptosis from intrinsic as well as extrinsic triggers by directly binding and inhibiting caspase-3, 7, 9[52]. Although many proteins could restrain upstream caspases, only XIAP have been shown an endogenous inhibitor of the terminal caspase cascade [53]. The overexpression of XIAP could protect CA1 hippocampal neurons and retinal ganglion cells from apoptosis in rat brain ischemia model and optic nerve axotomy model [53]. XIAP has been proposed as a therapeutic target for apoptosis-related diseases [54]. In addition to inhibiting apoptosis, XIAP was also involved in regulating inflammation, autophagy, ROS, and copper homeostasis, etc. [54]. Given that the powerful immunological and/or apoptosis-related regulatory functions of these factors, further research is needed to target these factors to engineer MenSCs for more prominent therapeutic effects.

KEGG analysis showed 55 factors and 42 factors involved in PI3K/AKT and MAPK signaling pathways, respectively. The disruption of PI3K/AKT and MAPK signaling pathway involved in the pathogenesis of neurodegenerative disorders including PD [55]. PI3K/Akt was reported as a target of natural products in the treatment of PD [56]. Several studies showed vitexin/ Icariin/amentoflavone could protect dopaminergic neurons against MPTP/MPP^+^ -induced apoptosis in mouse PD models and cell PD models by activating PI3K/Akt and MAPK signaling pathways [57–59]. Therefore, targeting PI3K/Akt and MAPK signaling pathway has been proposed as a reasonable approach to prevent the progression of PD [55, 56]. Further studies are needed to explore the functions of these two signaling pathways in the treatment of PD through activators or inhibitors related to these two pathways.

Except for naïve MSCs, genetically engineered MSCs [60], primed MSCs [61], MSCs-derived secretome/extracellular vesicles [62, 63], biomaterial-supported MSCs [64, 65], and the subpopulation of MSCs [66, 67] have been applied to enhance the therapeutic effects of MSCs in various diseases, which provides a new avenue for regenerative medicine. MSCs can be engineered to overexpress cytokine/growth factors with trophic, immunomodulation or reparative effects to accelerate endogenous tissue repair [60]. Pre‐treatment MSCs with chemical agents, starvation, hypoxia, inflammatory stimulation or 3D cultures has been reported to enhance the therapeutic effects after transplantation [61]. Cell-free therapies using soluble factors or extracellular vesicles secreted by MSCs could avoid immunological rejection [63]. Biomaterial scaffolds could provide a shelter for MSCs protecting them from the surrounding harsh microenvironment, achieve better engraftment, and promote cell survival [65, 68]. Multilineage differentiating stress-enduring (Muse) cells account for several percent in MSCs. Different from MSCs, Muse cells are pluripotent and positive for stage-specific embryonic antigen 3 (SSEA-3)[67]. Accumulating evidences have suggested that Muse cells had superior homing, stress-tolerance and differentiation ability than their parent MSCs. Thus, after transplantation, muse cells could achieve better therapeutic effect than MSCs [66, 67]. More research should be conducted to investigate the effects of the stem cell-based therapies mentioned above on PD, especially for muse cells, which has no application in PD yet.

In order to ensure normal homeostasis function in the brain, most animals use the blood–brain barrier (BBB) to effectively separate the nervous system from the circulatory system, ensuring metabolic and ion regulation in the brain, and also an additional barrier to prevent pathogens from invading the brain [69]. In vertebrates, microglia migrate to the developing nervous system to perform immune surveillance and remove excess cells and processes [2]. These local glial clearance mechanisms are usually adequate in healthy animals, but may become overloaded in the case of infection or during neurodegenerative and/or autoimmune diseases. Inflammatory conditions in the central nervous system trigger circulating lymphocytes and macrophages to leak through the BBB into the brain, clearing pathogens and cell debris, but may also lead to further brain damage [69]. It has been reported that autologous or donor macrophages could go across BBB and infiltrate to the degenerating brain of PD animal models [70, 71]. Considering this character, the engineered macrophages were employed as a drug delivery system to treat PD mice [70, 71]. From our in vitro studies, we can confirm that MenSCs reduced inflammation by regulating microglia polarization. However, our in vivo studies did not distinguish macrophage and microglia cells in the brain. With the WB and q-PCR results of M1 and M2 markers, we can only make a conclusion that MenSCs reduced neuroinflammation by shifting M1/M2 polarization. Further experiments need to be performed to reveal who is regulated by MenSCs, macrophages or microglia cells.

## Conclusions

In conclusion, MenSCs could alleviate neuroinflammation by modulating M1/M2 polarization, prevent the loss of dopaminergic neurons and improve motor function impairment in PD rats. We firstly demonstrated the biological process of factors secreted by MenSCs and the signal pathways involved in using protein array and bioinformatic analysis. The multifaceted functions of MenSCs are closely related to the soluble factors released by themselves.

## Supplementary Information

**Additional file 1**: **Table S1-S3. Table S1:** Quantitative RT-PCR primer sequences. **Table S2:** The results of GO TERM_BP enrichment analysis. **Table S3:** The results of KEGG_PATHWAY enrichment analysis.

**Additional file 2: Fig. S1**. The cell viability of BV2 and HAPI cells. A: BV2 cells were treated with various concentrations of 6-OHDA (0, 50, 100, 200, 300, 400 and 500 µM) for 24 h. B: HAPI cells were treated with various concentrations of 6-OHDA (0, 25, 50, 75, 100, 150, and 200 µM) for 24 h. Cell viability was measured using PrestoBlue reagent.

**Additional file 3: Fig. S2**. Uncropped WB images of Arg1, iNOS, TH and β-actin. A: WB result of Arg1 (36 kDa). B: WB result of iNOS (110-130 kDa, 65-70 kDa). C: WB result of β-actin (43 kDa). D: WB result of TH (57 kDa). E: WB result of β-actin (43 kDa). Dashed lines indicated where they were cropped to make Fig.6C and Fig.4J.

## Data Availability

The datasets generated during the current study are available in the Gene Expression Omnibus repository, ACCESSION NUMBER: GSE228520, GSE228521.

## Abbreviations

Akt: Serine-threonine kinase
Ang-1: Angiopoietin-1
Arg-1: Arginine-1
ARTN: Artemin
Bcl-2: B-cell lymphoma-2
BDNF: Brain-derived neurotrophic factor
CD11b/206: Cluster of differentiation antigen 11b/206
CDNF: Conserved dopamine neurotrophic factor
CSF: Cerebrospinal fluid
GDF-15: Growth differentiation factor 15
GDNF: Glial cell-derived neurotrophic factor
GO-BP: Gene Ontology Biological Process
HGF: Hepatocyte growth factor
IL-1α/2/4/6/10/13: Interleukin-1α/2/4/6/10/13
IFN-γ: Interferon-γ
IGF-1: Insulin-like growth factor-1
iNOS: Inducible nitric oxide synthase
KEGG: Kyoto Encyclopedia of Genes and Genomes
MANF: Mesencephalic astrocyte-derived neurotrophic factor
MCP-1: Monocyte chemoattractant protein-1
MenSCs: Menstrual blood-derived endometrial stem cells
MenSCs-CM: Conditioned medium of MenSCs
NGF: Nerve growth factor
NT-3/4/5: Neurotrophin-3/4/5
NTN: Neurturin
6-OHDA: 6-Hydroxydopamine
PD: Parkinson’s disease
PI3K: Phosphatidylinositol 3-kinase
PSPN: Persephin
SN: Substantia nigra
Str: Striatum
TH: Tyrosine hydroxylase
TGF-β: Transforming growth factor-β
TNF-α: Tumor necrosis factor-α
VTA: Ventral tegmental area
XIAP: X-linked inhibitor of apoptosis protein
YM1/2: Chitinase-like protein

## References

[1] Harms AS, Ferreira SA, Romero-Ramos M (2021). Periphery and brain, innate and adaptive immunity in Parkinson's disease. Acta Neuropathol.

[2] Song WM, Colonna M (2018). The identity and function of microglia in neurodegeneration. Nat Immunol.

[3] Ransohoff RM (2016). How neuroinflammation contributes to neurodegeneration. Science.

[4] Badanjak K, Fixemer S, Smajic S, Skupin A, Grunewald A (2021). The contribution of microglia to neuroinflammation in Parkinson's disease. Int J Mol Sci.

[5] Tang Y, Le W (2016). Differential roles of M1 and M2 microglia in neurodegenerative diseases. Mol Neurobiol.

[6] Guo S, Wang H, Yin Y (2022). Microglia polarization from M1 to M2 in neurodegenerative diseases. Front Aging Neurosci.

[7] Bartels T, De Schepper S, Hong S (2020). Microglia modulate neurodegeneration in Alzheimer's and Parkinson's diseases. Science.

[8] Ambrosi G, Kustrimovic N, Siani F, Rasini E, Cerri S, Ghezzi C (2017). Complex changes in the innate and adaptive immunity accompany progressive degeneration of the nigrostriatal pathway induced by intrastriatal injection of 6-Hydroxydopamine in the rat. Neurotox Res.

[9] Meng X, Ichim TE, Zhong J, Rogers A, Yin Z, Jackson J (2007). Endometrial regenerative cells: a novel stem cell population. J Transl Med.

[10] Cui CH, Uyama T, Miyado K, Terai M, Kyo S, Kiyono T (2007). Menstrual blood-derived cells confer human dystrophin expression in the murine model of Duchenne muscular dystrophy via cell fusion and myogenic transdifferentiation. Mol Biol Cell.

[11] Li H, Yahaya BH, Ng WH, Yusoff NM, Lin J (2019). Conditioned medium of human menstrual blood-derived endometrial stem cells protects against MPP(+)-induced cytotoxicity in vitro. Front Mol Neurosci.

[12] Xiang B, Chen L, Wang X, Zhao Y, Wang Y, Xiang C (2017). Transplantation of menstrual blood-derived mesenchymal stem cells promotes the repair of LPS-induced acute lung injury. Int J Mol Sci.

[13] Lv Y, Xu X, Zhang B, Zhou G, Li H, Du C (2014). Endometrial regenerative cells as a novel cell therapy attenuate experimental colitis in mice. J Transl Med.

[14] Chen X, Wu Y, Wang Y, Chen L, Zheng W, Zhou S (2020). Human menstrual blood-derived stem cells mitigate bleomycin-induced pulmonary fibrosis through anti-apoptosis and anti-inflammatory effects. Stem Cell Res Ther.

[15] Luz-Crawford P, Torres MJ, Noel D, Fernandez A, Toupet K, Alcayaga-Miranda F (2016). The immunosuppressive signature of menstrual blood mesenchymal stem cells entails opposite effects on experimental arthritis and graft versus host diseases. Stem Cells.

[16] Chen D, Zeng R, Teng G, Cai C, Pan T, Tu H (2021). Menstrual blood-derived mesenchymal stem cells attenuate inflammation and improve the mortality of acute liver failure combining with A2AR agonist in mice. J Gastroenterol Hepatol.

[17] Li Y, Gao H, Brunner TM, Hu X, Yan Y, Liu Y (2022). Menstrual blood-derived mesenchymal stromal cells efficiently ameliorate experimental autoimmune encephalomyelitis by inhibiting T cell activation in mice. Stem Cell Res Ther.

[18] Borlongan CV, Kaneko Y, Maki M, Yu SJ, Ali M, Allickson JG (2010). Menstrual blood cells display stem cell-like phenotypic markers and exert neuroprotection following transplantation in experimental stroke. Stem Cells Dev.

[19] Fathi-Kazerooni M, Fattah-Ghazi S, Darzi M, Makarem J, Nasiri R, Salahshour F (2022). Safety and efficacy study of allogeneic human menstrual blood stromal cells secretome to treat severe COVID-19 patients: clinical trial phase I & II. Stem Cell Res Ther.

[20] Dominici M, Le Blanc K, Mueller I, Slaper-Cortenbach I, Marini F, Krause D (2006). Minimal criteria for defining multipotent mesenchymal stromal cells. The International Society for Cellular Therapy position statement. Cytotherapy.

[21] Paxinos G, Watson C (2007). The rat brain in stereotaxic coordinates.

[22] Teixeira FG, Carvalho MM, Panchalingam KM, Rodrigues AJ, Mendes-Pinheiro B, Anjo S (2017). Impact of the secretome of human mesenchymal stem cells on brain structure and animal behavior in a rat model of Parkinson's disease. Stem Cell transl Med.

[23] Liu L, Duff K (2008). A technique for serial collection of cerebrospinal fluid from the cisterna magna in mouse. J Vis Exp.

[24] Dupont WD, Plummer WD (1998). Power and sample size calculations for studies involving linear regression. Control Clin Trials.

[25] Chaudhuri KR, Healy DG, Schapira AHV (2006). Non-motor symptoms of Parkinson's disease: diagnosis and management. Lancet Neurol.

[26] Carvalho MM, Campos FL, Coimbra B, Pêgo JM, Rodrigues C, Lima R (2013). Behavioral characterization of the 6-hydroxidopamine model of Parkinson's disease and pharmacological rescuing of non-motor deficits. Mol Neurodegener.

[27] Sousa N, Almeida OF, Wotjak CT (2006). A hitchhiker's guide to behavioral analysis in laboratory rodents. Genes Brain Behav.

[28] Andrzejewska A, Dabrowska S, Lukomska B, Janowski M (2021). Mesenchymal stem cells for neurological disorders. Adv Sci (Weinh).

[29] Staff NP, Jones DT, Singer W (2019). Mesenchymal stromal cell therapies for neurodegenerative diseases. Mayo Clin Proc.

[30] Boucherie C, Hermans E (2009). Adult stem cell therapies for neurological disorders: benefits beyond neuronal replacement?. J Neurosci Res.

[31] Wolff EF, Mutlu L, Massasa EE, Elsworth JD, Eugene Redmond D, Taylor HS (2015). Endometrial stem cell transplantation in MPTP- exposed primates: an alternative cell source for treatment of Parkinson's disease. J Cell Mol Med.

[32] Wolff EF, Gao XB, Yao KV, Andrews ZB, Du H, Elsworth JD (2011). Endometrial stem cell transplantation restores dopamine production in a Parkinson's disease model. J Cell Mol Med.

[33] McCoy MK, Martinez TN, Ruhn KA, Wrage PC, Keefer EW, Botterman BR (2008). Autologous transplants of Adipose-Derived Adult Stromal (ADAS) cells afford dopaminergic neuroprotection in a model of Parkinson's disease. Exp Neurol.

[34] Weiss ML, Medicetty S, Bledsoe AR, Rachakatla RS, Choi M, Merchav S (2006). Human umbilical cord matrix stem cells: preliminary characterization and effect of transplantation in a rodent model of Parkinson's disease. Stem Cells.

[35] Strelau J, Schober A, Sullivan A, Schilling L, Unsicker K (2003). Growth/differentiation factor-15 (GDF-15), a novel member of the TGF-beta superfamily, promotes survival of lesioned mesencephalic dopaminergic neurons in vitro and in vivo and is induced in neurons following cortical lesioning. J Neural Transm-Supp.

[36] Jiang WW, Zhang ZZ, He PP, Jiang LP, Chen JZ, Zhang XT (2021). Emerging roles of growth differentiation factor-15 in brain disorders (Review). Exp Ther Med.

[37] Varadarajan S, Breda C, Smalley JL, Butterworth M, Farrow SN, Giorgini F (2015). The transrepression arm of glucocorticoid receptor signaling is protective in mutant huntingtin-mediated neurodegeneration. Cell Death Differ.

[38] Machado V, Haas SJ, Von Bohlen Und Halbach O, Wree A, Krieglstein K, Unsicker K (2016). Growth/differentiation factor-15 deficiency compromises dopaminergic neuron survival and microglial response in the 6-hydroxydopamine mouse model of Parkinson's disease. Neurobiol Dis.

[39] Xiong WP, Yao WQ, Wang B, Liu K (2021). BMSCs-exosomes containing GDF-15 alleviated SH-SY5Y cell injury model of Alzheimer's disease via AKT/GSK-3beta/beta-catenin. Brain Res Bull.

[40] Thurston G, Rudge JS, Ioffe E, Papadopoulos N, Daly C, Vuthoori S (2005). The anti-inflammatory actions of angiopoietin-1. EXS.

[41] Sabirzhanov B, Faden AI, Aubrecht T, Henry R, Glaser E, Stoica BA (2018). MicroRNA-711-induced downregulation of angiopoietin-1 mediates neuronal cell death. J Neurotrauma.

[42] Huang ZW, Liu N, Li D, Zhang HY, Wang Y, Liu Y (2017). Angiopoietin-1 modified human umbilical cord mesenchymal stem cell therapy for endotoxin-induced acute lung injury in rats. Yonsei Med J.

[43] Yin Z, Gong G, Zhu C, Wang B, Sun C, Liu X (2020). Angiopoietin-1 protects neurons by inhibiting autophagy after neuronal oxygen-glucose deprivation/recovery injury. NeuroReport.

[44] Venkat P, Ning R, Zacharek A, Culmone L, Liang L, Landschoot-Ward J (2021). Treatment with an Angiopoietin-1 mimetic peptide promotes neurological recovery after stroke in diabetic rats. CNS Neurosci Ther.

[45] Torres MD, Garcia O, Tang C, Busciglio J (2018). Dendritic spine pathology and thrombospondin-1 deficits in Down syndrome. Free Radic Biol Med.

[46] Ghorbani S, Yong VW (2021). The extracellular matrix as modifier of neuroinflammation and remyelination in multiple sclerosis. Brain.

[47] Lu Z, Kipnis J (2010). Thrombospondin 1-a key astrocyte-derived neurogenic factor. FASEB J.

[48] Bray ER, Yungher BJ, Levay K, Ribeiro M, Dvoryanchikov G, Ayupe AC et al. Thrombospondin-1 mediates axon regeneration in retinal ganglion cells. Neuron. 2019; 103(4):642–57 e7. 10.1016/j.neuron.2019.05.044 PMC6706310 31255486

[49] Tian W, Sawyer A, Kocaoglu FB, Kyriakides TR (2011). Astrocyte-derived thrombospondin-2 is critical for the repair of the blood-brain barrier. Am J Pathol.

[50] Li Q, Fu X, Yuan J, Han S (2021). Contribution of thrombospondin-1 and -2 to lipopolysaccharide-induced acute respiratory distress syndrome. Mediators Inflamm.

[51] Muqri F, Helkin A, Maier KG, Gahtan V (2020). Thrombospondin-5 and fluvastatin promote angiogenesis and are protective against endothelial cell apoptosis. J Cell Biochem.

[52] Jost PJ, Vucic D (2020). Regulation of cell death and immunity by XIAP. Cold Spring Harb Perspect Biol.

[53] Holcik M, Gibson H, Korneluk RG (2001). XIAP: apoptotic brake and promising therapeutic target. Apoptosis.

[54] Hanifeh M, Ataei F. XIAP as a multifaceted molecule in Cellular Signaling. Apoptosis. 2022. 10.1007/s10495-022-01734-z 35661061

[55] Rai SN, Dilnashin H, Birla H, Singh SS, Zahra W, Rathore AS (2019). The role of PI3K/Akt and ERK in neurodegenerative disorders. Neurotox Res.

[56] Long HZ, Cheng Y, Zhou ZW, Luo HY, Wen DD, Gao LC (2021). PI3K/AKT signal pathway: a target of natural products in the prevention and treatment of Alzheimer's disease and Parkinson's disease. Front Pharmacol.

[57] Cao Q, Qin L, Huang F, Wang X, Yang L, Shi H (2017). Amentoflavone protects dopaminergic neurons in MPTP-induced Parkinson's disease model mice through PI3K/Akt and ERK signaling pathways. Toxicol Appl Pharmacol.

[58] Chen WF, Wu L, Du ZR, Chen L, Xu AL, Chen XH (2017). Neuroprotective properties of icariin in MPTP-induced mouse model of Parkinson's disease: Involvement of PI3K/Akt and MEK/ERK signaling pathways. Phytomedicine.

[59] Hu M, Li F, Wang W (2018). Vitexin protects dopaminergic neurons in MPTP-induced Parkinson's disease through PI3K/Akt signaling pathway. Drug Des Devel Ther.

[60] Kimbrel EA, Lanza R (2020). Next-generation stem cells - ushering in a new era of cell-based therapies. Nat Rev Drug Discov.

[61] Hu C, Wu Z, Li L (2020). Pre-treatments enhance the therapeutic effects of mesenchymal stem cells in liver diseases. J Cell Mol Med.

[62] Miceli V, Bulati M, Iannolo G, Zito G, Gallo A, Conaldi PG (2021). Therapeutic properties of mesenchymal stromal/stem cells: the need of cell priming for cell-free therapies in regenerative medicine. Int J Mol Sci.

[63] Phinney DG, Pittenger MF (2017). Concise review: MSC-derived exosomes for cell-free therapy. Stem Cells.

[64] Lv B, Zhang X, Yuan J, Chen Y, Ding H, Cao X (2021). Biomaterial-supported MSC transplantation enhances cell-cell communication for spinal cord injury. Stem Cell Res Ther.

[65] Khayambashi P, Iyer J, Pillai S, Upadhyay A, Zhang Y, Tran SD (2021). Hydrogel encapsulation of mesenchymal stem cells and their derived exosomes for tissue engineering. Int J Mol Sci.

[66] Park YJ, Niizuma K, Mokin M, Dezawa M, Borlongan CV (2020). Cell-based therapy for stroke: musing with Muse cells. Stroke.

[67] Simerman AA, Dumesic DA, Chazenbalk GD (2014). Pluripotent muse cells derived from human adipose tissue: a new perspective on regenerative medicine and cell therapy. Clin Transl Med.

[68] Murphy KC, Whitehead J, Zhou D, Ho SS, Leach JK (2017). Engineering fibrin hydrogels to promote the wound healing potential of mesenchymal stem cell spheroids. Acta Biomater.

[69] Zlokovic BV (2008). The blood-brain barrier in health and chronic neurodegenerative disorders. Neuron.

[70] Chen C, Guderyon MJ, Li Y, Ge G, Bhattacharjee A, Ballard C (2020). Non-toxic HSC transplantation-based macrophage/microglia-mediated GDNF delivery for Parkinson's disease. Mol Ther Methods Clin Dev.

[71] Zhao Y, Haney MJ, Jin YS, Uvarov O, Vinod N, Lee YZ (2019). GDNF-expressing macrophages restore motor functions at a severe late-stage, and produce long-term neuroprotective effects at an early-stage of Parkinson's disease in transgenic Parkin Q311X(A) mice. J Control Release.

