# Supplementary material for: Menstrual blood-derived endometrial stem cells alleviate neuroinflammation by modulating M1/M2 polarization in cell and rat Parkinson’s disease models
Source: Stem Cell Res Ther. 2023 Apr 13;14:85. doi: 10.1186/s13287-023-03330-7 (PMC10099022; doi:10.1186/s13287-023-03330-7)
Supplement: Supplementary file 1 — Additional file 1: Table S1-S3. Table S1: Quantitative RT-PCR primer sequences. Table S2: The results of GO TERM_BP enrichment analysis. Table S3: The results of KEGG_PATHWAY enrichment analysis. [file 13287_2023_3330_MOESM1_ESM.docx]

**Table 1 Quantitative RT-PCR Primer Sequences**

| **Gene name** | **Forward primer sequence (5' → 3')** | **Reverse primer sequence (5' → 3')** |
| --- | --- | --- |
| m-GAPDH | GTTGTCTCCTGCGACTTCA | TGGTCCAGGGTTTCTTACTC |
| m-IL-1β | GCCTCGTGCTGTCGGACCCATAT | TCCTTTGAGGCCCAAGGCCACA |
| m-IL-6 | TAGTCCTTCCTACCCCAATTTCC | TTGGTCCTTAGCCACTCCTTC |
| m-IL-10 | GCTCTTACTGACTGGCATGAG | CGCAGCTCTAGGAGCATGTG |
| m-iNOS | GCTCGCTTTGCCACGGACGA | AAGGCAGCGGGCACATGCAA |
| m-TGF-β | TGATACGCCTGAGTGGCTGTCT | CACAAGAGCAGTGAGCGCTGAA |
| m-TNF-α | CCCTCCTGGCCAACGGCATG | TCGGGGCAGCCTTGTCCCTT |
| Rat-GAPDH | TGACTGGAAGAGCGGAGAGTACT | TTCGGGCTGATGTACCAGTTC |
| Rat-Arg-1 | AGCCAATGAAGAGCTGGCTGGT | AACTGCCAGACTGTGGTCTCCA |
| Rat-CD11b | GAGCAGCACTGAGATCCTGTTTAA | ATACGACTCCTGCCCTGGAA |
| Rat-CD206 | TCAGCTATTGGACGCGAGGCA | TCCGGGTTGCAAGTTGCCGT |
| Rat-IL-1β | CCCTGAACTCAACTGTGAAATAGCA | CCCAAGTCAAGGGCTTGGAA |
| Rat-IL-6 | ATTGTATGAACAGCGATGATGCAC | CCAGGTAGAAACGGAACTCCAGA |
| Rat-IL-10 | CGGCTGAGGCGCTGT | TGCCTTGCTCTTATTTTCACAGG |
| Rat-iNOS | GACGAGACGGATAGGCAGAG | GTGGGGTTGTTGCTGAACTT |
| Rat-MHCⅡ | ACAGGAATTGTGTCCACGGG | AAGGCCTGGGTCAGGGATAA |
| Rat-TGFβ | CACCCGCGTGCTAATGGT | GGCACTGCTTCCCGAATG |
| Rat-TNFα | GCCACCACGCTCTTCTGTC | GCTACGGGCTTGTCACTCG |
| Rat-YM1/2 | TACTCACTTCCACAGGAGCAGG | CTCCAGTGTAGCCATCCTTAGG |

**Supplementary Table 2 The results of GO TERM_BP enrichment analysis**

| **No.** | **ID** | **Description** | **Count** | **P.Value** | **Genes** |
| --- | --- | --- | --- | --- | --- |
| 1 | GO:0008284 | positive regulation of cell proliferation | 76 | 6.88E-37 | IL21/CNTFR/CSF3/CNTF/FLT1/CSF2/EPO/SIRPG/PRL/FGF2/CXCL5/CX3CL1/CRKL/FGF5/ESM1/FGF7/NAMPT/KDR/CTSH/ITGAV/TIMP1/IL6R/PDGFRB/IL11/IL15/FSHB/FLT3LG/NRG1/MIF/OSMR/PGF/EREG/CDC25B/IFNG/KIT/IL6ST/EPHA1/CTF1/PDGFB/PDGFA/THBS1/EGFR/INS/DPP4/GRK5/ERBB2/NTF3/PDGFC/S100A13/FGF20/CGA/TGFB2/TGFB1/EGF/NTRK3/OSM/IL31RA/IGF2/VEGFC/IGF1/IL2/BMP5/VEGFA/FGF17/CXCL10/BST1/GDF9/IL6/IL7/TNFSF4/IL9/MDM2/PTPN6/FOLR2/IL7R/LGMN |
| 2 | GO:0006954 | inflammatory response | 64 | 6.31E-34 | CXCL6/IL1RN/CXCL8/IL23R/CXCL1/IL1RAP/CXCL3/TNF/CXCL2/CXCL5/CX3CL1/IL18RAP/IL36B/OLR1/LGALS9/TNFRSF4/IL15/IGFBP4/IL1R1/IL13/MIF/TNFRSF1B/TNFRSF1A/TLR1/IL1A/MEP1B/IL23A/KIT/CHST1/CHI3L1/S100A8/CHST2/EPHA2/CRP/CALCA/SEMA7A/CCL11/THBS1/CCL8/CCL7/CCL5/CCL4/SPP1/CCL3/CCL2/CD14/CCL19/CCL18/CCL23/TGFB1/RARRES2/IL36G/CXCL10/CXCL11/IL6/IL5/AXL/TNFSF4/IL9/SDC1/PTX3/CDO1/FOLR2/IL17B |
| 3 | GO:0007165 | signal transduction | 101 | 7.87E-29 | CNTFR/IL1RN/CNTF/EPO/FGF2/CXCL16/CRKL/FGF7/ALCAM/PLAU/GRAP2/NAMPT/ICOSLG/PDGFRB/IGFBP1/IL4R/IGFBP4/IGFBP2/FLT3LG/PGF/BCAM/IL23A/CD226/CD274/LHB/DAPP1/TNFRSF11B/CSF2RB/KLRK1/FGF20/IGFBP6/TNFSF18/PSPN/TNFSF12/IGF1/IGF2R/FGF17/CXCL10/BST1/CXCL11/TG/ARTN/TNFSF4/IL9/IL7R/FGF12/RET/ACVRL1/IL21/CXCL6/CXCL8/IL25/CXCL1/CXCL5/MDK/TIMP1/CD34/NCK1/IL10/IL11/DFFA/IL15/IFNGR1/IL13/PLAUR/RGMB/TNFRSF1A/TLR1/CD200R1/KIT/ANGPTL3/LTA/MET/TNFRSF21/GRN/CCL11/GRP/EGFR/TYMP/CCL8/CCL7/GPNMB/ERBB2/NTF3/CCL4/SPP1/CCL2/CCL18/CCL23/ANGPT2/IL31/ERAP2/GDF15/RYK/TIE1/MOK/VEGFC/AXL/FAS/LTBR/IL17B |
| 4 | GO:0030335 | positive regulation of cell migration | 46 | 5.66E-27 | RET/CD274/GRN/SEMA7A/FLT1/CCL11/PDGFB/PDGFA/THBS1/EGFR/CXCL16/INS/CCL7/GPNMB/PLAU/MDK/CCL5/PDGFC/NTF3/CCL3/KDR/CTSH/ITGAV/CGA/SEMA6B/PDGFRB/TGFB1/MMP7/F10/EGF/HGF/MMP2/NTRK3/FSHB/IGF1/F3/VEGFA/FGR/F7/KIT/ANGPTL3/NUMB/ADAM9/ITGA5/EPHA1/EPHA2 |
| 5 | GO:0007267 | cell-cell signaling | 43 | 6.44E-26 | CXCL6/CALCA/CTF1/LHB/SIRPG/PDGFA/CXCL5/CX3CL1/INS/FGF5/CCL8/EFNB3/CCL7/GPNMB/CCL5/GRAP2/CCL4/NAMPT/NTF3/CCL3/FGF20/CCL18/TSHB/TNFSF18/CCL23/IL15/GDF15/SH2D1A/IL36G/INHBA/MERTK/IL2/PGF/AGT/EREG/FGF17/CXCL10/CXCL11/IL7/LTA/FGF12/SIGLEC6/IL17B |
| 6 | GO:0006955 | immune response | 57 | 1.44E-23 | IL21/CXCL6/CSF3/IL1RN/CSF2/CXCL8/IL1RAP/CTSV/TNF/CXCL2/CXCL5/CX3CL1/CTSS/IL18RAP/IL36B/CTSL/FTH1/CTLA4/B2M/TNFRSF4/IL10/IL4R/IL15/IL1R1/IL13/PDCD1LG2/TNFRSF1B/TLR1/IL1A/LTA/CD274/SEMA7A/CCL11/THBS1/CCL8/CCL5/CCL4/CCL3/CCL2/TNFRSF14/CCL19/CCL18/CCL23/TNFSF12/OSM/IL2/IL5/IL7/TNFSF4/IL9/FAS/LTBR/ULBP2/IL7R/IL17B/BMPR1A/NECTIN1 |
| 7 | GO:0007155 | cell adhesion | 60 | 3.39E-23 | SIGLEC9/SIRPG/ICAM2/MSLN/CX3CL1/LOXL2/ICAM1/ADAMTSL1/COMP/CDH4/ALCAM/BOC/BSG/ITGAV/CD34/EDIL3/EPHB4/POSTN/ITGA1/OMD/RGMB/BCAN/BCAM/CDH11/ADAM9/CD226/ITGA5/EPHA2/ENG/CCL11/LAMA4/AMIGO2/CD99L2/PSEN1/THBS2/NID2/THBS1/DPP4/ACAN/GPNMB/ADAM23/CCL4/SPP1/CCL2/NCAM1/JAM3/MUC16/MOG/SIGLEC11/IL2/SELL/FAP/CNTN1/CNTN3/TGFBI/FOLR2/NECTIN4/SIGLEC7/SIGLEC6/NECTIN1 |
| 8 | GO:0014068 | positive regulation of phosphatidylinositol 3-kinase signaling | 27 | 7.15E-23 | CSF3/FLT1/PDGFB/PDGFA/TREM2/FGF2/TNF/INS/CCL5/PDGFC/KDR/PDGFRB/TGFB2/ANGPT1/EGF/HGF/NTRK3/OSM/IGF1/DCN/AGT/VEGFA/FGR/GH1/KIT/CD28/PTPN6 |
| 9 | GO:0042531 | positive regulation of tyrosine phosphorylation of STAT protein | 25 | 1.89E-22 | IL21/CNTF/CTF1/CSF2/EPO/IL23R/TNF/GHR/IFNL1/CCL5/IL6R/TNFSF18/IL15/IL13/OSM/IL31RA/IGF1/IL2/TNFRSF1A/GH1/IL6/IFNG/IL23A/KIT/IL6ST |
| 10 | GO:0043410 | positive regulation of MAPK cascade | 32 | 3.30E-20 | RET/FLT1/PDGFB/PDGFA/FGF2/TNF/CX3CL1/INS/SOX2/PRDX2/ERBB2/KDR/TIMP2/IGFBP6/IL6R/IL11/EGF/IGFBP4/GDF15/RYK/HGF/IGFBP3/NTRK3/ITGA1/OSM/IGF2/FRS2/IGF1/MMP8/VEGFA/IL6/KIT |
| 11 | GO:0070374 | positive regulation of ERK1 and ERK2 cascade | 36 | 3.13E-19 | SEMA7A/CCL11/EPO/PDGFB/PDGFA/TREM2/FGF2/TNF/EGFR/CX3CL1/ICAM1/CRKL/CCL8/CCL7/GPNMB/CCL5/CCL4/PDGFC/CCL3/KDR/FGF20/CCL2/CCL19/LGALS9/CCL18/PDGFRB/CCL23/TGFB1/ANGPT1/NRG1/GCG/IGF1/MIF/VEGFA/IL1A/CHI3L1 |
| 12 | GO:0030593 | neutrophil chemotaxis | 24 | 3.68E-19 | CXCL6/CCL23/TGFB2/CCL11/CXCL8/ITGA1/CXCL1/CXCL3/CXCL2/CXCL5/CX3CL1/CXCL10/LGALS3/CXCL11/CCL8/CCL7/CCL5/BSG/CCL4/CCL3/CCL2/CCL19/CCL18/S100A8 |
| 13 | GO:0050731 | positive regulation of peptidyl-tyrosine phosphorylation | 25 | 9.08E-19 | CSF3/PDGFB/TREM2/ICAM1/GHR/FGF7/NTF3/TNFRSF14/IL6R/IL11/TGFB1/ANGPT1/IL15/HGF/OSM/IGF2/IGF1/MIF/AGT/VEGFA/GH1/IL6/IL5/CNTN1/ITGA5 |
| 14 | GO:0051897 | positive regulation of protein kinase B signaling | 28 | 9.21E-19 | RET/CSF3/PDGFA/FGF2/THBS1/TNF/EGFR/CX3CL1/INS/CCL3/CCL19/TGFB1/ANGPT1/F10/EGF/GDF15/OSM/IGF2/NRG1/F3/VEGFA/F7/LIN28A/AXL/CD28/CHI3L1/MET/ENG |
| 15 | GO:0019221 | cytokine-mediated signaling pathway | 30 | 1.82E-18 | CNTFR/CSF3/IL23R/IL20RA/CSF2RB/CX3CL1/IL22RA2/GHR/IL36B/CCL2/IL6R/IFNAR2/IL4R/IFNGR1/IL31RA/IL36G/OSMR/IL17RD/F3/EREG/TNFRSF1A/IL22RA1/IL1A/IL6/IL5/IL7/KIT/PTPN6/IL6ST/IL7R |
| 16 | GO:0010628 | positive regulation of gene expression | 51 | 3.09E-18 | RET/CNTF/CSF2/SPI1/CXCL8/TREM2/FGF2/TNF/FGF5/FGF7/IL36B/CTSH/LGALS9/CD34/IFNGR1/FSHB/MST1/IL13/NRG1/NGF/MMP8/DKK1/F3/EDAR/IL1A/IFNG/ENG/CRP/PDGFB/PSEN1/INS/ERBB2/FGF20/CCL3/LDLR/TGFB1/ANGPT1/EGF/NOS3/NTRK3/IL36G/IGF1/INHBA/VEGFA/FGF17/IL6/CD28/LCN2/MDM2/CNTN1/IL7R |
| 17 | GO:0071222 | cellular response to lipopolysaccharide | 32 | 9.50E-18 | CD274/CXCL6/CSF3/CSF2/CXCL8/SERPINE1/CXCL1/BCL10/CXCL3/TFPI/TNF/CXCL2/CXCL5/ICAM1/KLRK1/ADAMTS13/IL36B/CCL2/CD14/B2M/IL10/IL36G/PDCD1LG2/TNFRSF1B/IL1A/CXCL10/CXCL11/IL6/AXL/TNFSF4/LCN2/ADAM9 |
| 18 | GO:0050918 | positive chemotaxis | 17 | 9.41E-16 | ANGPT1/HGF/PDGFB/VEGFC/MIF/FGF2/CX3CL1/PGF/VEGFA/CXCL10/LGALS3/FGF7/GPNMB/CCL5/NTF3/CCL3/MET |
| 19 | GO:0043406 | positive regulation of MAP kinase activity | 21 | 1.03E-15 | PDGFRB/TGFB1/FLT1/EGF/NTRK3/PDGFB/PDGFA/MIF/PSEN1/FGF2/THBS1/TNF/EGFR/VEGFA/GHR/GH1/PDGFC/KIT/NTF3/ERBB2/ADAM9 |
| 20 | GO:0002548 | monocyte chemotaxis | 17 | 1.54E-15 | CCL23/CALCA/FLT1/CCL11/PDGFB/CX3CL1/LGALS3/IL6/CCL8/CCL7/CCL5/CCL4/CCL3/CCL2/CCL19/CCL18/IL6R |
| 21 | GO:0033674 | positive regulation of kinase activity | 20 | 2.27E-15 | EPHB6/PDGFRB/RET/FLT1/RYK/TIE1/NTRK3/TREM2/BCL10/MERTK/EGFR/IL2/AXL/KIT/ERBB2/KDR/EPHA1/MET/EPHB4/EPHA2 |
| 22 | GO:0051781 | positive regulation of cell division | 17 | 7.10E-15 | TGFB2/TGFB1/PDGFB/OSM/IGF2/VEGFC/PDGFA/FGF2/PGF/SIRT2/EREG/VEGFA/FGF5/IL1A/FGF7/MDK/PDGFC |
| 23 | GO:0046718 | viral entry into host cell | 22 | 1.02E-14 | SCARB2/MOG/EGFR/ICAM1/DPP4/EFNB3/LAMP1/CTSL/AXL/BSG/ITGAV/NCAM1/TNFRSF14/ITGA5/NECTIN4/LDLR/TNFRSF4/CLEC4G/NECTIN2/CTSB/EPHA2/NECTIN1 |
| 24 | GO:0070098 | chemokine-mediated signaling pathway | 19 | 1.59E-14 | CXCL6/CCL23/CCL11/CXCL8/CXCL1/CXCL3/CXCL2/CXCL5/CX3CL1/CXCL10/CXCL11/CCL8/CCL7/CCL5/CCL4/CCL3/CCL2/CCL19/CCL18 |
| 25 | GO:0007169 | transmembrane receptor protein tyrosine kinase signaling pathway | 24 | 3.08E-14 | EPHB6/PDGFRB/RET/FLT1/RYK/BDNF/TIE1/NTRK3/IL31RA/FRS2/NRG1/NGF/MERTK/EGFR/FGR/AXL/KIT/NTF3/ERBB2/KDR/EPHA1/MET/EPHB4/EPHA2 |
| 26 | GO:0045766 | positive regulation of angiogenesis | 26 | 5.66E-14 | ACVRL1/GRN/FLT1/CCL11/CXCL8/SERPINE1/FGF2/THBS1/KDR/CTSH/CD34/ANGPT2/NOS3/HGF/TIE1/TNFSF12/VEGFC/F3/PGF/VEGFA/IL1A/ANGPTL3/CHI3L1/ANGPTL4/EPHA1/ENG |
| 27 | GO:0006935 | chemotaxis | 23 | 6.40E-14 | CXCL6/CCL23/CCL11/CXCL8/RARRES2/PLAUR/CXCL1/FGF2/CXCL2/CXCL5/CX3CL1/TYMP/CXCL16/CXCL10/CXCL11/CCL8/CCL7/PLAU/CCL5/CCL3/CCL2/LGALS9/CCL18 |
| 28 | GO:0001525 | angiogenesis | 31 | 1.44E-13 | ACVRL1/FLT1/CXCL8/SERPINE1/PDGFA/TYMP/ESM1/BSG/KDR/CCL2/ITGAV/EPHB4/JAM3/PDGFRB/ANGPT2/ANGPT1/EGF/NOS3/MMP2/TIE1/TNFSF12/EREG/VEGFA/FAP/ANGPTL3/ANG/TGFBI/ITGA5/ANGPTL4/EPHA1/ENG |
| 29 | GO:0042102 | positive regulation of T cell proliferation | 17 | 9.79E-13 | IL21/CD274/IL15/IL23R/TNFRSF13C/PDCD1LG2/VTCN1/IL6/CD6/IL23A/CCL5/TNFSF4/CD28/CCL19/IL6ST/NCK1/CD276 |
| 30 | GO:0006508 | proteolysis | 38 | 1.06E-12 | CFD/PRSS1/HTRA2/PLG/KLK3/KLK7/CTSS/DPP4/ACAN/CASP7/DPP7/ADAMTS13/PLAU/CTSL/ADAM23/CTSH/OLR1/CTSB/MMP7/MME/ERAP2/F10/MMP1/MMP2/MST1/MMP3/MMP8/PGC/MMP10/MMP12/MEP1B/FAP/PAPPA/LCN1/REN/TPP1/MASP1/LGMN |
| 31 | GO:0001934 | positive regulation of protein phosphorylation | 27 | 2.27E-12 | SEMA7A/TREM2/FGF2/TNF/EGFR/CRKL/FGF5/FGF7/GPNMB/ERBB2/KDR/FGF20/TGFB1/ANGPT1/HGF/RARRES2/NTRK3/PLAUR/IGF2/VEGFC/PGF/VEGFA/FGF17/IFNG/CD6/FAS/ENG |
| 32 | GO:0048661 | positive regulation of smooth muscle cell proliferation | 17 | 3.38E-12 | PDGFRB/NOTCH3/TGFB1/MMP2/IL13/PDGFB/IGF1/FGF2/THBS1/TNF/EGFR/CX3CL1/EREG/IL6/CCL5/NAMPT/IL6R |
| 33 | GO:0050729 | positive regulation of inflammatory response | 20 | 6.40E-12 | TNFSF18/IL21/IL15/SERPINE1/OSM/TNF/EGFR/IL2/CX3CL1/AGT/TNFRSF1A/LGALS1/FABP4/IFNG/MDK/IL23A/TNFSF4/CCL3/LDLR/S100A8 |
| 34 | GO:0018108 | peptidyl-tyrosine phosphorylation | 22 | 7.21E-12 | EPHB6/PDGFRB/RET/FLT1/RYK/EGF/TIE1/NTRK3/PDGFB/MERTK/EGFR/FGR/IL5/AXL/KIT/ERBB2/KDR/PTPN6/EPHA1/MET/EPHB4/EPHA2 |
| 35 | GO:0007275 | multicellular organism development | 26 | 3.33E-11 | EPHB6/RET/CSF3/FLT1/FSTL1/EGFR/FSTL3/ERBB2/KDR/EPHB4/PDGFRB/RYK/TIE1/FST/NTRK3/OSM/IGF2/KREMEN2/MERTK/AXL/KIT/CDH11/EPHA1/MET/CHST2/EPHA2 |
| 36 | GO:0071356 | cellular response to tumor necrosis factor | 21 | 9.65E-11 | CCL23/POSTN/CALCA/CCL11/CXCL8/THBS1/CX3CL1/ICAM1/CCL8/ADAMTS13/FABP4/CCL7/CCL5/CCL4/CCL3/LCN2/CCL2/CHI3L1/CCL19/CCL18/TNFRSF21 |
| 37 | GO:0043066 | negative regulation of apoptotic process | 40 | 1.14E-10 | TNFRSF6B/AMIGO2/XIAP/PSEN1/VTCN1/THBS1/FSTL1/EGFR/CX3CL1/COMP/PRDX2/GRK5/KDR/CTSH/TIMP1/IL10/TNFSF18/PDGFRB/NQO1/ANGPT1/HGF/PLAUR/IL31RA/GCG/IGF1/MIF/DKK1/IL2/VEGFA/FABP1/IL6/AXL/IL7/CEACAM5/MDM2/CD28/FAS/CD27/ANGPTL4/IL6ST |
| 38 | GO:0048247 | lymphocyte chemotaxis | 12 | 1.40E-10 | CCL23/CCL8/CCL11/CCL7/CCL5/CCL4/CCL3/CCL2/CCL19/CCL18/CX3CL1/CXCL16 |
| 39 | GO:0043491 | protein kinase B signaling | 14 | 1.42E-10 | TGFB1/NRG1/IGF1/MERTK/FGF2/TNF/SIRT2/AXL/CCL5/CCL3/CD28/KDR/CCL2/EPHA2 |
| 40 | GO:0042060 | wound healing | 18 | 1.76E-10 | PDGFRB/TGFB2/POSTN/TGFB1/SDC4/PDGFA/NRG1/IGF1/FGF2/DCN/TSKU/EGFR/INS/EREG/ERBB2/SDC1/S100A8/ENG |
| 41 | GO:0071347 | cellular response to interleukin-1 | 17 | 1.92E-10 | CCL23/CCL11/CXCL8/MMP2/TFPI/CX3CL1/ICAM1/CCL8/CCL7/CCL5/CCL4/CCL3/LCN2/CCL2/CHI3L1/CCL19/CCL18 |
| 42 | GO:0022617 | extracellular matrix disassembly | 13 | 3.36E-10 | PRSS1/MMP7/MMP1/MMP2/MMP3/PLG/CTSV/MMP8/CTSS/KLK7/MMP10/MMP12/ENG |
| 43 | GO:0007568 | aging | 23 | 6.96E-10 | IL10/PDGFRB/IGFBP1/NQO1/CALCA/TGFB1/MMP7/MME/EPO/IL15/MMP2/IGFBP2/HTRA2/TNFRSF1B/FGF2/DCN/CX3CL1/AGT/LOXL2/CASP7/NAMPT/TIMP2/TIMP1 |
| 44 | GO:0001666 | response to hypoxia | 22 | 1.24E-09 | ACVRL1/TGFB2/POSTN/TGFB1/ANGPT2/EPO/MMP2/VEGFC/PDGFA/THBS1/TNF/PGF/LOXL2/VEGFA/DPP4/IL1A/F7/PLAU/LTA/ANG/ANGPTL4/ENG |
| 45 | GO:0048245 | eosinophil chemotaxis | 9 | 1.42E-09 | LGALS3/CCL8/CCL11/CCL7/CCL5/CCL4/CCL3/CCL2/CX3CL1 |
| 46 | GO:0097191 | extrinsic apoptotic signaling pathway | 13 | 1.58E-09 | TGFB2/TGFB1/TNFSF12/INHBA/TNFRSF1B/TNF/PRDX2/CTTN/IFNG/IL7/FAS/CD27/IL6R |
| 47 | GO:0001938 | positive regulation of endothelial cell proliferation | 15 | 1.95E-09 | IL10/ACVRL1/BMPR2/CCL11/EGF/TNFSF12/PDGFB/VEGFC/FGF2/F3/PGF/VEGFA/FGF7/KDR/ANG |
| 48 | GO:0001819 | positive regulation of cytokine production | 14 | 2.64E-09 | IL10/IL21/IL15/MIF/TNF/AGT/INS/EREG/FGR/IL1A/IFNG/TNFSF4/S100A13/CD28 |
| 49 | GO:0061844 | antimicrobial humoral immune response mediated by antimicrobial peptide | 17 | 3.59E-09 | CXCL6/CALCA/CCL11/CXCL8/CXCL1/CXCL3/CXCL2/CXCL5/CXCL10/LGALS3/CXCL11/CCL8/GNLY/ANG/CCL19/CCL18/B2M |
| 50 | GO:0002639 | positive regulation of immunoglobulin production | 10 | 5.09E-09 | IL10/IL21/IL6/RBP4/IL4R/IL5/TNFSF4/IL13/TNFRSF4/IL2 |
| 51 | GO:0046427 | positive regulation of JAK-STAT cascade | 11 | 7.03E-09 | IL10/GHR/GH1/IL6/IFNL1/IL5/CCL5/KIT/PRL/IL7R/TNF |
| 52 | GO:0007166 | cell surface receptor signaling pathway | 27 | 9.48E-09 | RTN4R/CD274/CTF1/SIGLEC9/PRL/EGFR/ERBB2/BSG/CCL2/TNFRSF14/CD14/FCRL1/IFNAR2/IL1R1/INHBA/MIF/PDCD1LG2/MERTK/AGT/CXCL10/IFNG/PAPPA/CD28/CD27/IL7R/EPHA1/MET |
| 53 | GO:0010862 | positive regulation of pathway-restricted SMAD protein phosphorylation | 12 | 1.31E-08 | ACVRL1/GDF9/TGFB2/TGFB1/BMPR2/GDF15/BMP8A/GDF3/INHBA/BMPR1A/ENG/BMP5 |
| 54 | GO:0045840 | positive regulation of mitotic nuclear division | 10 | 1.46E-08 | PDGFRB/IL1A/EGF/PDGFB/IGF2/CD28/IGF1/TNF/EREG/INS |
| 55 | GO:0098609 | cell-cell adhesion | 21 | 1.51E-08 | KIRREL3/CSTA/ITGA1/ICAM2/PSEN1/EGFR/CX3CL1/LRP6/ICAM1/LGALS1/BOC/CDH11/CNTN1/DSG1/ITGAV/DSG3/ITGA5/CD34/JAM2/JAM3/NECTIN1 |
| 56 | GO:0033138 | positive regulation of peptidyl-serine phosphorylation | 15 | 1.85E-08 | IL11/CSF3/TGFB1/ANGPT1/BDNF/NTRK3/OSM/GCG/MIF/NGF/TNF/EGFR/VEGFA/IL6/NTF3 |
| 57 | GO:0042327 | positive regulation of phosphorylation | 10 | 2.02E-08 | EGF/CCL5/ANG/BCL10/MIF/PSEN1/THBS1/EGFR/EREG/VEGFA |
| 58 | GO:0031295 | T cell costimulation | 11 | 2.08E-08 | DPP4/CD274/KLRK1/LGALS1/EFNB3/CD28/PTPN6/TNFRSF14/TNFRSF13C/CCL19/PDCD1LG2 |
| 59 | GO:0032729 | positive regulation of interferon-gamma production | 14 | 2.60E-08 | IL21/IL1R1/IL23R/TNF/IL2/KLRK1/IFNL1/IL23A/TNFSF4/LTA/CD226/CD14/LGALS9/CD276 |
| 60 | GO:0007411 | axon guidance | 20 | 4.34E-08 | EPHB6/SEMA6B/RET/NOTCH3/ROBO3/SEMA7A/RYK/BDNF/UNC5C/ADAMTSL1/CDH4/EFNB3/ARTN/BOC/BSG/CNTN1/EPHA1/EPHB4/EPHA2/NECTIN1 |
| 61 | GO:0010629 | negative regulation of gene expression | 26 | 4.83E-08 | ACVRL1/SPI1/CXCL8/PDGFB/PSEN1/FGF2/TNF/INS/CRKL/CCL3/KDR/LGALS9/LDLR/CD34/TGFB2/TGFB1/IGF1/MIF/MMP8/AGT/VEGFA/IFNG/CD28/LGMN/BMPR1A/ENG |
| 62 | GO:0071346 | cellular response to interferon-gamma | 15 | 5.58E-08 | CCL23/CCL11/TNF/CX3CL1/ICAM1/CCL8/ADAMTS13/CCL7/CCL5/CCL4/CCL3/CCL2/CCL19/LGALS9/CCL18 |
| 63 | GO:0016477 | cell migration | 24 | 6.06E-08 | PDGFRB/TGFB2/TGFB1/FLT1/SDC4/MMP2/MERTK/THBS1/CRKL/AXL/GPC1/KDR/GPC3/SDC1/ADAM9/ITGAV/GPC5/ANG/IGFBP6/MET/ENG/EPHA2/NCK1/JAM3 |
| 64 | GO:0001501 | skeletal system development | 17 | 8.96E-08 | TGFB2/FST/IGF2/MEPE/PRELP/TNFRSF11B/GDF3/IGF1/FGFRL1/BMP5/COMP/ACAN/BCAN/FRZB/CDH11/MATN3/EPHA2 |
| 65 | GO:0030509 | BMP signaling pathway | 14 | 9.83E-08 | ACVRL1/TGFB2/TGFB1/BMPR2/GDF15/FST/BMP8A/GDF3/RGMB/BMP5/COMP/GDF9/ENG/BMPR1A |
| 66 | GO:0042130 | negative regulation of T cell proliferation | 11 | 1.05E-07 | IL10/CD274/TGFB1/SDC4/GPNMB/CTLA4/PTPN6/PDCD1LG2/VTCN1/TNFRSF21/CLEC4G |
| 67 | GO:0032760 | positive regulation of tumor necrosis factor production | 15 | 1.70E-07 | CD84/IFNGR1/MIF/PSEN1/MMP8/THBS1/TLR1/IL1A/IL6/IFNG/IL23A/CCL3/CD14/CCL19/LGALS9 |
| 68 | GO:0031663 | lipopolysaccharide-mediated signaling pathway | 10 | 1.81E-07 | SPI1/TGFB1/CD6/NOS3/CCL5/CCL3/CCL2/CD14/BCL10/TNF |
| 69 | GO:0007157 | heterophilic cell-cell adhesion via plasma membrane cell adhesion molecules | 11 | 2.35E-07 | CDH4/ALCAM/CD6/AMIGO1/CEACAM5/AMIGO2/ITGA5/NECTIN4/SCARF2/ICAM1/NECTIN1 |
| 70 | GO:0010951 | negative regulation of endopeptidase activity | 17 | 2.42E-07 | CSTB/CSTA/TFPI2/SERPINE1/CRIM1/NGF/TFPI/SERPINB8/AGT/SERPINB6/SPINT1/CD109/TIMP2/LCN1/TIMP1/PI3/TIMP4 |
| 71 | GO:0030198 | extracellular matrix organization | 18 | 2.50E-07 | POSTN/MMP7/MMP1/MMP2/MMP3/TNFRSF11B/MMP8/TNF/MMP10/MMP12/ADAMTSL1/PRDX4/ADAMTS13/SPINT1/PTX3/TGFBI/MATN3/MATN2 |
| 72 | GO:0032733 | positive regulation of interleukin-10 production | 10 | 2.87E-07 | CD274/IL6/IL23A/HGF/TNFSF4/IL13/CD28/TREM2/LGALS9/CD34 |
| 73 | GO:0030574 | collagen catabolic process | 10 | 2.87E-07 | MMP12/MMP7/MMP1/CTSL/MMP2/MMP3/MMP8/CTSS/MMP10/CTSB |
| 74 | GO:0051384 | response to glucocorticoid | 12 | 3.72E-07 | IL10/GHR/IL6/IL1RN/MDK/PAPPA/IGFBP2/ADAM9/SDC1/CTSV/CDO1/TNF |
| 75 | GO:0031640 | killing of cells of other organism | 12 | 5.04E-07 | LGALS3/CXCL10/CXCL11/CCL8/CCL11/CXCL8/GNLY/CXCL1/CCL19/CXCL3/CCL18/CXCL2 |
| 76 | GO:0043524 | negative regulation of neuron apoptotic process | 17 | 7.12E-07 | IL10/CNTFR/GRN/CNTF/ANGPT1/BDNF/PSEN1/NGF/THAP11/MDK/AXL/NTF3/FGF20/KDR/CCL2/IL6ST/LGMN |
| 77 | GO:0008285 | negative regulation of cell proliferation | 30 | 8.88E-07 | ACVRL1/CXCL8/SIRPG/PLG/CXCL1/FGF2/THBS1/FGFRL1/IFNL1/GPNMB/FRZB/FTH1/TIMP2/IGFBP6/IL10/TGFB2/CCL23/TGFB1/NOS3/IGFBP3/ITGA1/OSM/INHBA/NGF/SIRT2/BMP5/EREG/IL1A/IL6/PTPN6 |
| 78 | GO:0006959 | humoral immune response | 11 | 1.12E-06 | BST1/IL6/IFNG/IL7/SH2D1A/LTA/CD28/CCL2/TREM2/TNF/TNFRSF21 |
| 79 | GO:0042493 | response to drug | 23 | 1.17E-06 | IL10/RET/NQO1/TGFB2/TGFB1/IGFBP2/VEGFC/TNFRSF11B/INHBA/COMT/THBS1/PGF/ICAM1/LGALS1/MDK/MDM2/TIMP2/LTA/LCN2/FOSB/B2M/TIMP4/ENG |
| 80 | GO:0032740 | positive regulation of interleukin-17 production | 8 | 1.40E-06 | IL21/IL6/TGFB1/IL15/IL23A/IL23R/OSM/IL2 |
| 81 | GO:0050830 | defense response to Gram-positive bacterium | 15 | 1.54E-06 | CRP/CALCA/RARRES2/TNF/FGR/IL6/KLRK1/LTA/ACP5/ANG/TNFRSF14/IL7R/IL6R/B2M/EPHA2 |
| 82 | GO:0043536 | positive regulation of blood vessel endothelial cell migration | 10 | 1.71E-06 | TGFB1/ANGPT1/NOS3/PDGFB/KDR/VEGFC/PLG/THBS1/FGF2/VEGFA |
| 83 | GO:0007565 | female pregnancy | 13 | 1.92E-06 | TGFB1/FSHB/IGFBP2/PRL/COMT/PGF/AGT/EPYC/PAPPA/NAMPT/FOSB/LGALS9/ITGA5 |
| 84 | GO:0016485 | protein processing | 12 | 2.25E-06 | COMP/F7/CASP7/KEL/ADAMTS13/MME/ADAM9/CPE/PSEN1/F3/CTSS/METAP2 |
| 85 | GO:0050714 | positive regulation of protein secretion | 10 | 2.41E-06 | IL1A/TGFB2/TGFB1/IL13/VEGFC/ADAM9/TREM2/ANG/IGF1/INS |
| 86 | GO:0010595 | positive regulation of endothelial cell migration | 11 | 3.12E-06 | GRN/BMPR2/ANGPT1/EGF/BSG/LCN2/KDR/THBS1/FGF2/AGT/VEGFA |
| 87 | GO:0042104 | positive regulation of activated T cell proliferation | 8 | 3.13E-06 | EPO/IL23A/IL23R/IGFBP2/IGF2/IGF1/IL2/ICOSLG |
| 88 | GO:0007259 | JAK-STAT cascade | 9 | 3.44E-06 | GHR/IFNAR2/GH1/CSF2/IFNL1/IFNG/IL31RA/CCL2/CSF2RB |
| 89 | GO:0009887 | animal organ morphogenesis | 15 | 3.44E-06 | IGF2/VEGFC/PDGFA/FGF2/DCN/EREG/FGF5/FGF17/COMP/TMEFF1/FGF7/IL7/PDGFC/FGF20/CCL2 |
| 90 | GO:0033209 | tumor necrosis factor-mediated signaling pathway | 10 | 3.92E-06 | TNFSF18/TNFRSF19/FAS/TNFRSF14/TNFRSF13C/TNFRSF1B/TNF/TNFRSF4/EDA2R/TNFRSF1A |
| 91 | GO:1904707 | positive regulation of vascular smooth muscle cell proliferation | 10 | 3.92E-06 | IL10/MMP2/MDM2/PDGFB/FRS2/IGF1/TNF/FGF2/AGT/BMPR1A |
| 92 | GO:0001774 | microglial cell activation | 8 | 4.01E-06 | TLR1/IFNG/IFNGR1/NAMPT/IL13/TREM2/TNF/CX3CL1 |
| 93 | GO:0032755 | positive regulation of interleukin-6 production | 13 | 4.08E-06 | IL1RAP/BCL10/MMP8/TNF/EREG/TLR1/IL1A/IL6/IFNG/TNFSF4/BSG/LGALS9/IL6R |
| 94 | GO:0098586 | cellular response to virus | 12 | 4.10E-06 | MMP12/IL21/IFNAR2/CXCL10/IL6/IFNL1/IFNG/IFNGR1/CCL5/CCL19/LGALS9/LGALS8 |
| 95 | GO:0048146 | positive regulation of fibroblast proliferation | 10 | 5.33E-06 | PDGFRB/TGFB1/PDGFC/PDGFB/PDGFA/IGF1/MIF/EGFR/AGT/EREG |
| 96 | GO:0071363 | cellular response to growth factor stimulus | 10 | 6.18E-06 | ACVRL1/BMPR2/ANGPT2/ERBB2/MDM2/PDGFB/HTRA2/TNFRSF1B/THBS1/BMPR1A |
| 97 | GO:0007159 | leukocyte cell-cell adhesion | 8 | 6.37E-06 | NT5E/CALCA/SELL/CCL5/OLR1/ITGA5/JAM2/ICAM1 |
| 98 | GO:0043552 | positive regulation of phosphatidylinositol 3-kinase activity | 8 | 6.37E-06 | PDGFRB/FGR/TGFB1/FLT1/KIT/PDGFB/CCL19/FGF2 |
| 99 | GO:0045785 | positive regulation of cell adhesion | 10 | 7.14E-06 | TNFSF18/ANGPT1/MDK/CCL5/ERBB2/ITGAV/NRG1/NID1/TNF/VEGFA |
| 100 | GO:0030890 | positive regulation of B cell proliferation | 9 | 7.16E-06 | IL21/BST1/IL5/IL7/IL13/TNFRSF13C/MIF/TNFRSF4/IL2 |
| 101 | GO:0009410 | response to xenobiotic stimulus | 19 | 1.17E-05 | IL10/RET/TGFB2/MMP2/IGFBP2/VEGFC/PDGFA/TNFRSF11B/INHBA/ENO2/THBS1/TNF/PGF/MDK/LTA/SLITRK5/FOSB/TIMP4/ENG |
| 102 | GO:1902895 | positive regulation of pri-miRNA transcription from RNA polymerase II promoter | 9 | 1.18E-05 | IL10/TGFB2/SPI1/TGFB1/PDGFB/PRL/TNF/FGF2/BMPR1A |
| 103 | GO:0051603 | proteolysis involved in cellular protein catabolic process | 9 | 1.38E-05 | PSMA1/FAP/CTSL/MDM2/CTSH/CTSV/CTSS/CTSB/LGMN |
| 104 | GO:1901215 | negative regulation of neuron death | 10 | 1.42E-05 | GHR/CSF3/CD200R1/GPNMB/EPO/IL13/HTRA2/ENO2/TNFRSF1B/CD34 |
| 105 | GO:0032496 | response to lipopolysaccharide | 15 | 1.43E-05 | EPO/NOS3/IL23R/IL13/CSF2RB/COMT/DCN/PRDX2/CD6/LTA/ACP5/REN/LGALS9/S100A8/TIMP4 |
| 106 | GO:0032757 | positive regulation of interleukin-8 production | 10 | 1.61E-05 | TLR1/IL6/CALCA/SERPINE1/CHI3L1/CD14/BCL10/LGALS9/TNF/F3 |
| 107 | GO:0034097 | response to cytokine | 9 | 1.88E-05 | CD274/TIMP2/ACP5/TIMP1/IL6ST/OSMR/IL6R/TIMP4/CXCL16 |
| 108 | GO:0032526 | response to retinoic acid | 9 | 2.17E-05 | PDGFRB/RBP4/TIE1/MMP2/IGFBP2/PDGFA/CTSH/DKK1/IGF2R |
| 109 | GO:0071260 | cellular response to mechanical stimulus | 11 | 2.92E-05 | TGFB1/MMP7/IL13/FAS/BCL10/LTBR/FGF2/EGFR/AGT/ENG/TNFRSF1A |
| 110 | GO:1901224 | positive regulation of NIK/NF-kappaB signaling | 10 | 2.96E-05 | EDAR/IL23A/CD27/TREM2/CD14/CCL19/LGALS9/MMP8/TNF/EGFR |
| 111 | GO:0007399 | nervous system development | 25 | 3.15E-05 | CNTFR/SEMA7A/CXCL1/FGF2/FGF5/EFNB3/MDK/BOC/NTF3/SEMA6B/PSPN/BDNF/NTRK3/CRIM1/NRG1/INHBA/MERTK/VEGFA/FGF17/AXL/CNTN3/TPP1/MDGA2/MET/FGF12 |
| 112 | GO:0070371 | ERK1 and ERK2 cascade | 9 | 3.31E-05 | CCL11/EGF/KDR/CTSH/ITGAV/IGF1/LGALS9/FGF2/AGT |
| 113 | GO:0007160 | cell-matrix adhesion | 12 | 3.38E-05 | BCAM/ADAMTS13/ITGA1/ANGPTL3/ADAM9/ITGAV/ITGA5/MSLN/NID1/CD34/NID2/JAM3 |
| 114 | GO:0050679 | positive regulation of epithelial cell proliferation | 10 | 3.72E-05 | GRN/FGF7/TGFB1/ERBB2/IGF1/FGF2/EGFR/BMPR1A/BMP5/VEGFA |
| 115 | GO:0043627 | response to estrogen | 10 | 3.72E-05 | PDGFRB/F7/IL4R/MME/EPO/MMP2/IGFBP2/TNFRSF11B/COMT/TSHB |
| 116 | GO:0001942 | hair follicle development | 8 | 4.19E-05 | EDAR/TGFB2/CD109/TNFRSF19/PDGFA/INHBA/DKK1/EGFR |
| 117 | GO:0007156 | homophilic cell adhesion via plasma membrane adhesion molecules | 15 | 4.27E-05 | KIRREL3/RET/ROBO3/CD84/AMIGO1/AMIGO2/CDH4/CEACAM5/BSG/CDH11/DSG1/DSG3/NECTIN4/NECTIN2/NECTIN1 |
| 118 | GO:0030324 | lung development | 11 | 4.32E-05 | FGF7/RBP4/MME/NOS3/GPC3/CHI3L1/FGF2/EGFR/FSTL3/BMPR1A/VEGFA |
| 119 | GO:0030307 | positive regulation of cell growth | 11 | 4.75E-05 | IGFBP1/TGFB2/ERBB2/IL9/NRG1/EXTL3/EGFR/IL2/S100A8/INS/CXCL16 |
| 120 | GO:0008543 | fibroblast growth factor receptor signaling pathway | 9 | 4.91E-05 | FGF17/FGF5/FGF7/FGF20/FRS2/FGF12/FGF2/FGFRL1/CRKL |
| 121 | GO:0032355 | response to estradiol | 12 | 5.17E-05 | GHR/PDGFRB/GH1/NQO1/F7/POSTN/TGFB1/IGFBP2/PDGFA/ENO2/TFPI/AGT |
| 122 | GO:0050829 | defense response to Gram-negative bacterium | 11 | 5.72E-05 | IL22RA1/IL6/CALCA/IL23A/IL23R/RARRES2/SERPINE1/TREM2/TNFRSF14/IL6R/B2M |
| 123 | GO:0032720 | negative regulation of tumor necrosis factor production | 10 | 6.38E-05 | IL10/GPNMB/AXL/ACP5/PTPN6/TREM2/IGF1/LGALS9/CD34/CX3CL1 |
| 124 | GO:1904646 | cellular response to beta-amyloid | 8 | 8.85E-05 | NAMPT/LCN2/TREM2/IGF1/PSEN1/TNF/ICAM1/LGMN |
| 125 | GO:0060395 | SMAD protein signal transduction | 9 | 1.01E-04 | GDF9/TGFB2/TGFB1/GDF15/BMP8A/GDF3/INHBA/AFP/BMP5 |
| 126 | GO:0050673 | epithelial cell proliferation | 8 | 1.16E-04 | EGF/HGF/KIT/GPC3/KDR/PSEN1/EGFR/BMPR1A |
| 127 | GO:0050766 | positive regulation of phagocytosis | 8 | 1.33E-04 | IL15RA/IFNG/IL15/SIRPG/TREM2/PTX3/MERTK/TNF |
| 128 | GO:0048013 | ephrin receptor signaling pathway | 8 | 1.51E-04 | EPHB6/EFNB3/NTRK3/MMP2/EPHA1/EPHB4/EPHA2/NCK1 |
| 129 | GO:0006915 | apoptotic process | 30 | 1.61E-04 | TNFRSF6B/EPO/TNFRSF11B/BCL10/PSEN1/COMP/CASP7/LGALS1/GRK5/CTSH/CD14/RELT/IGFBP3/TNFSF12/TNFRSF19/UNC5C/TNFRSF1A/EDAR/IL1A/IFNG/STK17A/CEACAM5/LTA/LCN2/MDM2/CHI3L1/FAS/LTBR/S100A8/TNFRSF21 |
| 130 | GO:0000165 | MAPK cascade | 12 | 1.73E-04 | RET/TGFB1/IGFBP4/CCL5/IGFBP3/IL31RA/CCL3/CCL2/PTPN6/NRG1/TNF/EGFR |
| 131 | GO:0009615 | response to virus | 11 | 2.31E-04 | FGR/IFNAR2/CCL8/CCL11/IFNG/IFNGR1/CCL5/TNFSF4/CCL4/CCL19/TNF |
| 132 | GO:0045471 | response to ethanol | 12 | 2.43E-04 | NQO1/CSF3/RBP4/NTRK3/IL13/CD27/CD14/CDO1/TNF/IL2/S100A8/ICAM1 |
| 133 | GO:0031334 | positive regulation of protein complex assembly | 8 | 2.44E-04 | LGALS3/TGFB1/IFNG/MMP1/MMP3/NRG1/TNF/VEGFA |
| 134 | GO:0050852 | T cell receptor signaling pathway | 11 | 2.49E-04 | MOG/CD28/CTLA4/PTPN6/BCL10/VTCN1/PSEN1/TNFRSF21/ICOSLG/CRKL/CD276 |
| 135 | GO:0000187 | activation of MAPK activity | 9 | 2.56E-04 | RET/GDF15/KIT/ITGA1/NRG1/IGFBP6/IGF1/FGF2/AGT |
| 136 | GO:0002244 | hematopoietic progenitor cell differentiation | 9 | 3.08E-04 | TGFB1/FLT1/FST/KIT/KDR/PTPN6/INHBA/PSEN1/FSTL3 |
| 137 | GO:0051092 | positive regulation of NF-kappaB transcription factor activity | 13 | 3.19E-04 | TNFSF18/TGFB1/IL1RAP/BCL10/TNF/CX3CL1/EDA2R/AGT/INS/ICAM1/IL18RAP/LGALS9/S100A8 |
| 138 | GO:0043547 | positive regulation of GTPase activity | 14 | 4.01E-04 | RTN4R/CCL23/CCL11/CX3CL1/ICAM1/CCL8/CCL7/CCL5/CCL4/ERBB2/CCL3/CCL2/CCL19/CCL18 |
| 139 | GO:0046330 | positive regulation of JNK cascade | 10 | 4.09E-04 | EDAR/IL1A/TNFRSF19/XIAP/CD27/LTBR/CCL19/MMP8/TNF/EDA2R |
| 140 | GO:0006952 | defense response | 9 | 4.41E-04 | CST3/CXCL6/CD84/IL31RA/INHBA/CXCL2/CXCL5/CX3CL1/ICOSLG |
| 141 | GO:0043123 | positive regulation of I-kappaB kinase/NF-kappaB signaling | 14 | 4.90E-04 | CANT1/TNFRSF19/BCL10/TNF/CX3CL1/EDA2R/TNFRSF1A/EDAR/IL1A/LGALS1/S100A13/LTBR/CCL19/LGALS9 |
| 142 | GO:0007179 | transforming growth factor beta receptor signaling pathway | 10 | 5.11E-04 | ACVRL1/GDF9/TGFB2/SPI1/TGFB1/GDF15/FSHB/ADAM9/BMPR1A/ENG |
| 143 | GO:0009612 | response to mechanical stimulus | 8 | 5.69E-04 | POSTN/ANGPT2/MMP2/IGFBP2/FOSB/CHI3L1/THBS1/DCN |
| 144 | GO:0043065 | positive regulation of apoptotic process | 19 | 5.85E-04 | PDGFRB/DFFA/TGFB1/MMP2/IGFBP3/TNFRSF10C/HTRA2/UNC5C/BCL10/PSEN1/TNF/IGF2R/IL6/LGALS1/STK17A/FRZB/LTA/FAS/CTLA4 |
| 145 | GO:0001649 | osteoblast differentiation | 11 | 6.00E-04 | SOX2/SEMA7A/BMPR2/GPNMB/IGFBP3/SPP1/IGF2/CCL3/FGF2/BMPR1A/EPHA2 |
| 146 | GO:0016525 | negative regulation of angiogenesis | 11 | 6.38E-04 | CXCL10/TGFB2/ANGPT2/TIE1/KLK3/PRL/THBS2/THBS1/DCN/AGT/EPHA2 |
| 147 | GO:0048812 | neuron projection morphogenesis | 8 | 6.89E-04 | KIRREL3/CTTN/UBB/BDNF/ITGA1/NTF3/NGF/EGFR |
| 148 | GO:0001503 | ossification | 9 | 7.22E-04 | CALCA/SORT1/CDH11/SPP1/BMP8A/ACP5/EGFR/FSTL3/BMP5 |
| 149 | GO:0010976 | positive regulation of neuron projection development | 11 | 7.22E-04 | SCARB2/RET/MDK/BDNF/EPO/NTRK3/AMIGO1/CNTN1/AGT/CX3CL1/NCK1 |
| 150 | GO:0043154 | negative regulation of cysteine-type endopeptidase activity involved in apoptotic process | 8 | 9.87E-04 | FABP1/HGF/MDM2/XIAP/CD27/THBS1/TNF/VEGFA |
| 151 | GO:0008584 | male gonad development | 10 | 0.001079 | PDGFRB/TGFB2/PRDX4/LHB/KIT/PDGFA/REN/INHBA/FSTL3/CRKL |
| 152 | GO:0060326 | cell chemotaxis | 8 | 0.00117 | PDGFRB/HGF/KIT/PDGFB/CX3CL1/EPHA2/ENG/CRKL |
| 153 | GO:0045944 | positive regulation of transcription from RNA polymerase II promoter | 45 | 0.00137 | ACVRL1/NOTCH3/CSF3/BMPR2/SPI1/IL25/PSIP1/FGF2/TNF/EGFR/CX3CL1/LRP6/FSTL3/SOX2/NAMPT/CGA/NCK1/IL10/IL11/TGFB1/LUM/HGF/FSHB/OSM/IGF2/IGF1/INHBA/SIRT2/DCN/IL2/BMP5/TNFRSF1A/VEGFA/THAP11/MMP12/IL1A/CXCL10/IL6/IL23A/MDM2/CD28/FOSB/MET/BMPR1A/ENG |
| 154 | GO:0002250 | adaptive immune response | 22 | 0.001722 | TNFSF18/CD274/CD84/ERAP2/SH2D1A/TNFRSF13C/BCL10/PDCD1LG2/VTCN1/IL2/CTSS/KLRK1/ALCAM/IFNG/CD6/CTSL/CTSH/CTLA4/TNFRSF14/TNFRSF21/ICOSLG/JAM3 |
| 155 | GO:0045669 | positive regulation of osteoblast differentiation | 8 | 0.001883 | IL6/BMPR2/HGF/IGF1/IL6ST/IL6R/FGF2/BMPR1A |
| 156 | GO:0030154 | cell differentiation | 29 | 0.002049 | SEMA7A/SPI1/FGF2/FSTL1/EGFR/FSTL3/TYMP/SOX2/FGF5/FGF7/KLRK1/MDK/FRZB/FGF20/SEMA6B/ANGPT1/RARRES2/FST/TNFSF12/NTRK3/BMP8A/NRG1/INHBA/PGF/VEGFA/FGR/EDAR/FGF17/PTPN6 |
| 157 | GO:0001933 | negative regulation of protein phosphorylation | 8 | 0.002347 | TGFB1/ANGPT1/NTRK3/CD109/IGFBP3/IL2/CRKL/LRP6 |
| 158 | GO:0045087 | innate immune response | 26 | 0.002838 | CRP/CD84/CALCA/BCL10/IL1RAP/LGALS3/IFNL1/IL36B/TNFRSF14/PI3/CD14/B2M/RARRES2/SH2D1A/IL36G/MIF/SIRT2/FGR/TLR1/CD6/IL23A/AXL/LCN2/ANG/PTX3/S100A8 |
| 159 | GO:0006468 | protein phosphorylation | 23 | 0.002854 | RET/ACVRL1/SEMA7A/TGFB2/CALCA/TGFB1/BMPR2/CCL11/CSF2/IGFBP3/PDGFB/MOK/CSF2RB/MERTK/CDC25B/FGR/GRK5/STK17A/CD109/ERBB2/CCL2/ST3GAL1/BMPR1A |
| 160 | GO:0030334 | regulation of cell migration | 9 | 0.003551 | FGF17/FGF5/FGF7/TGFB1/LAMA4/FGF20/UNC5C/FGF2/NCK1 |
| 161 | GO:0007229 | integrin-mediated signaling pathway | 9 | 0.003755 | FGR/SEMA7A/ADAMTS13/ITGA1/ADAM23/ANGPTL3/ADAM9/ITGAV/ITGA5 |
| 162 | GO:0006874 | cellular calcium ion homeostasis | 9 | 0.003755 | CCL23/KEL/TGFB1/CCL8/CCL11/CCL7/CCL5/CCL3/CCL19 |
| 163 | GO:0007613 | memory | 8 | 0.003769 | BDNF/NTF3/TREM2/ITGA5/PSEN1/NGF/SORCS3/LGMN |
| 164 | GO:0009636 | response to toxic substance | 8 | 0.003769 | PDGFRB/NQO1/ADAMTS13/CCL5/CCL4/MDM2/CCL3/SDC1 |
| 165 | GO:0007596 | blood coagulation | 8 | 0.004016 | F7/F10/PLAU/TFPI2/PLAUR/PLG/TFPI/F3 |
| 166 | GO:0042127 | regulation of cell proliferation | 11 | 0.004534 | CXCL10/TGFB2/CXCL11/TGFB1/BMPR2/IL4R/PLAU/KIT/XIAP/AGT/ENG |
| 167 | GO:0090090 | negative regulation of canonical Wnt signaling pathway | 10 | 0.006181 | SOX2/IGFBP1/MDK/FRZB/IGFBP4/IGFBP2/GPC3/IGFBP6/DKK1/DKK3 |
| 168 | GO:0007417 | central nervous system development | 10 | 0.007664 | SEMA6B/ACAN/BCAN/MOG/PSPN/PDGFC/ADAM23/TIMP2/TPP1/TIMP4 |
| 169 | GO:0008360 | regulation of cell shape | 10 | 0.009403 | FGR/CCL11/CCL7/KIT/CCL3/KDR/CCL2/COCH/ICAM1/VEGFA |
| 170 | GO:0042981 | regulation of apoptotic process | 13 | 0.009408 | DFFA/XIAP/FRS2/BCL10/AGT/CXCL10/PRDX2/LGALS1/NTF3/FAS/GAS1/PTPN6/CTSB |
| 171 | GO:0007507 | heart development | 12 | 0.011861 | ACVRL1/ACAN/IL1A/TGFB2/CASP7/RBP4/TGFB1/NTRK3/MMP2/ERBB2/PDGFB/FGF12 |
| 172 | GO:0051726 | regulation of cell cycle | 15 | 0.014369 | TGFB2/TGFB1/CXCL8/MOK/XIAP/INHBA/MIF/FGF2/THBS1/SIRT2/SOX2/IFNG/FAP/GRK5/MDM2 |
| 173 | GO:0046777 | protein autophosphorylation | 10 | 0.023596 | PDGFRB/FGR/FLT1/GRK5/ERBB2/KIT/KDR/EPHA1/EPHB4/EGFR |
| 174 | GO:0045893 | positive regulation of transcription, DNA-templated | 26 | 0.024086 | RET/ACVRL1/EPO/PDGFB/BCL10/PSEN1/FGF2/TNF/EGFR/LRP6/SOX2/FGF7/IFNL1/NUP85/MDK/SPP1/IL10/TGFB1/EGF/IL31RA/IGF1/INHBA/RGMB/AGT/IL6/IL5 |
| 175 | GO:0030308 | negative regulation of cell growth | 8 | 0.026738 | ACVRL1/GDF9/TGFB2/TGFB1/BMPR2/FRZB/INHBA/AGT |
| 176 | GO:0050728 | negative regulation of inflammatory response | 8 | 0.028792 | IL10/NT5E/HGF/IL13/ACP5/IL2/IL22RA2/TNFRSF1A |

**Supplementary Table 3 The results of KEGG_PATHWAY enrichment analysis**

| **No.** | **ID** | **Description** | **Count** | **P.Value** | **Genes** |
| --- | --- | --- | --- | --- | --- |
| 1 | hsa04060 | Cytokine-cytokine receptor interaction | 99 | 4.52E-67 | CNTFR/CSF3/IL1RN/CNTF/TNFRSF6B/CSF2/EPO/IL23R/IL1RAP/TNF/CXCL16/IL18RAP/IL36B/TNFRSF4/IL6R/IFNAR2/IL15RA/IL4R/IL1R1/EDAR/IL23A/IL6ST/IL20RA/TNFRSF11B/CSF2RB/TNFRSF14/TNFSF18/TGFB2/TGFB1/TNFSF12/IL31RA/TNFRSF10C/IL36G/INHBA/GDF3/IL2/BMP5/IL22RA1/CXCL10/GDF9/GH1/CXCL11/IL6/IL5/IL7/TNFSF4/IL9/CD27/IL7R/BMPR1A/ACVRL1/IL21/CXCL6/BMPR2/CXCL8/IL25/CXCL1/TNFRSF13C/PRL/CXCL3/CXCL2/CXCL5/CX3CL1/EDA2R/GHR/IL10/IL11/IL15/IFNGR1/IL13/TNFRSF19/NGF/OSMR/TNFRSF1B/TNFRSF1A/IL1A/IFNG/LTA/TNFRSF21/CTF1/CCL11/CCL8/IFNL1/CCL7/CCL5/CCL4/CCL3/CCL2/CCL19/CCL18/RELT/CCL23/IL31/GDF15/OSM/BMP8A/FAS/LTBR/IL17B |
| 2 | hsa04630 | JAK-STAT signaling pathway | 42 | 1.94E-22 | IL21/CNTFR/CSF3/CNTF/CTF1/CSF2/EPO/IL23R/IL20RA/PDGFB/PDGFA/CSF2RB/PRL/EGFR/IL22RA2/GHR/IFNL1/IL6R/IL10/PDGFRB/IFNAR2/IL11/IL15RA/IL4R/IFNGR1/IL15/EGF/IL13/OSM/OSMR/IL2/IL22RA1/GH1/IL6/IL5/IFNG/IL23A/IL7/IL9/PTPN6/IL6ST/IL7R |
| 3 | hsa04061 | Viral protein interaction with cytokine and cytokine receptor | 34 | 3.24E-22 | CXCL6/CCL11/CXCL8/IL20RA/CXCL1/CXCL3/TNF/CXCL2/CXCL5/CX3CL1/CCL8/IL18RAP/CCL7/CCL5/CCL4/CCL3/CCL2/TNFRSF14/CCL19/CCL18/IL6R/IL10/CCL23/TNFRSF10C/TNFRSF1B/IL2/TNFRSF1A/IL22RA1/CXCL10/CXCL11/IL6/LTA/LTBR/IL6ST |
| 4 | hsa04151 | PI3K-Akt signaling pathway | 55 | 2.43E-18 | CSF3/FLT1/EPO/PRL/FGF2/GHR/COMP/FGF5/FGF7/KDR/ITGAV/IL6R/PDGFRB/IFNAR2/IL4R/HGF/ITGA1/FLT3LG/NGF/OSMR/PGF/EREG/KIT/ITGA5/MET/EPHA2/LAMA4/PDGFB/PDGFA/THBS2/THBS1/EGFR/INS/ERBB2/NTF3/PDGFC/SPP1/FGF20/ANGPT2/ANGPT1/EGF/NOS3/BDNF/OSM/IGF2/VEGFC/IGF1/IL2/VEGFA/FGF17/GH1/IL6/IL7/MDM2/IL7R |
| 5 | hsa05323 | Rheumatoid arthritis | 29 | 8.30E-18 | CXCL6/FLT1/CSF2/CXCL8/CXCL1/CXCL3/TNF/CXCL2/CXCL5/ICAM1/CTSL/CCL5/CCL3/CCL2/CTLA4/ACP5/IL11/TGFB2/TGFB1/ANGPT1/IL15/MMP1/MMP3/VEGFA/IL1A/IL6/IFNG/IL23A/CD28 |
| 6 | hsa04657 | IL-17 signaling pathway | 24 | 1.13E-12 | CXCL6/CSF3/CCL11/CXCL8/CSF2/IL25/MMP1/IL13/MMP3/CXCL1/CXCL3/CXCL2/TNF/CXCL5/CXCL10/IL6/IL5/IFNG/CCL7/LCN2/CCL2/FOSB/S100A8/IL17B |
| 7 | hsa04010 | MAPK signaling pathway | 42 | 1.14E-12 | FLT1/PDGFB/PDGFA/IL1RAP/FGF2/TNF/EGFR/INS/CRKL/FGF5/FGF7/ERBB2/PDGFC/NTF3/KDR/FGF20/CD14/PDGFRB/TGFB2/TGFB1/ANGPT2/ANGPT1/EGF/IL1R1/BDNF/HGF/IGF2/VEGFC/FLT3LG/IGF1/NGF/PGF/EREG/TNFRSF1A/CDC25B/VEGFA/FGF17/IL1A/KIT/FAS/MET/EPHA2 |
| 8 | hsa05200 | Pathways in cancer | 57 | 8.69E-12 | RET/SPI1/CXCL8/EPO/IL23R/FGF2/CRKL/FGF5/CASP7/FGF7/ITGAV/IL6R/PDGFRB/IFNAR2/IL15RA/IL4R/IFNGR1/IL15/MMP1/HGF/MMP2/IL13/FLT3LG/PGF/IFNG/IL23A/KIT/IL6ST/MET/NOTCH3/LAMA4/PDGFB/PDGFA/XIAP/CSF2RB/KLK3/EGFR/LRP6/ERBB2/FGF20/NQO1/TGFB2/TGFB1/EGF/IGF2/VEGFC/IGF1/AGT/IL2/VEGFA/FGF17/IL6/IL5/IL7/MDM2/FAS/IL7R |
| 9 | hsa05144 | Malaria | 17 | 4.03E-11 | IL10/CSF3/TGFB2/TGFB1/CXCL8/HGF/THBS2/THBS1/TNF/ICAM1/COMP/IL6/KLRK1/IFNG/CCL2/SDC1/MET |
| 10 | hsa04514 | Cell adhesion molecules | 27 | 4.55E-10 | CD274/SDC4/ICAM2/CD99L2/VTCN1/ICAM1/CDH4/ALCAM/SLITRK5/CTLA4/NCAM1/ITGAV/CD34/ICOSLG/CD99/JAM2/JAM3/CD276/PDCD1LG2/SELL/CD6/CD28/CNTN1/SDC1/CD226/NECTIN2/NECTIN1 |
| 11 | hsa05205 | Proteoglycans in cancer | 30 | 2.03E-09 | SDC4/FGF2/THBS1/TNF/EGFR/PLAU/CTSL/GPC1/ERBB2/KDR/GPC3/ITGAV/TGFB2/TGFB1/LUM/HGF/MMP2/PLAUR/IGF2/FRS2/IGF1/DCN/VEGFA/CTTN/MDM2/FAS/SDC1/PTPN6/ITGA5/MET |
| 12 | hsa04640 | Hematopoietic cell lineage | 20 | 8.90E-09 | IL11/CSF3/IL4R/CSF2/MME/IL1R1/EPO/ITGA1/FLT3LG/TNF/IL1A/IL6/IL5/IL7/KIT/CD14/ITGA5/IL7R/IL6R/CD34 |
| 13 | hsa04668 | TNF signaling pathway | 21 | 1.32E-08 | CXCL6/CSF2/IL15/MMP3/VEGFC/CXCL1/CXCL3/TNFRSF1B/CXCL2/TNF/CXCL5/CX3CL1/ICAM1/TNFRSF1A/CXCL10/CASP7/IL6/CCL5/LTA/FAS/CCL2 |
| 14 | hsa05321 | Inflammatory bowel disease | 16 | 2.50E-08 | IL10/IL21/TGFB2/TGFB1/IL4R/IFNGR1/IL23R/IL13/TNF/IL2/IL1A/IL6/IL18RAP/IL5/IFNG/IL23A |
| 15 | hsa01521 | EGFR tyrosine kinase inhibitor resistance | 17 | 6.15E-08 | PDGFRB/EGF/HGF/PDGFB/PDGFA/NRG1/IGF1/FGF2/EGFR/VEGFA/IL6/AXL/PDGFC/ERBB2/KDR/MET/IL6R |
| 16 | hsa04064 | NF-kappa B signaling pathway | 19 | 1.18E-07 | CXCL8/IL1R1/XIAP/CXCL1/TNFRSF13C/BCL10/CXCL3/CXCL2/TNF/EDA2R/ICAM1/TNFRSF1A/EDAR/PLAU/CCL4/LTA/CD14/LTBR/CCL19 |
| 17 | hsa04014 | Ras signaling pathway | 29 | 1.73E-07 | FLT1/PDGFB/PDGFA/FGF2/EGFR/INS/FGF5/FGF7/PDGFC/NTF3/KDR/FGF20/PDGFRB/ANGPT2/ANGPT1/EGF/BDNF/HGF/IGF2/VEGFC/FLT3LG/IGF1/NGF/PGF/VEGFA/FGF17/KIT/MET/EPHA2 |
| 18 | hsa04015 | Rap1 signaling pathway | 27 | 2.27E-07 | FLT1/PDGFB/PDGFA/FGF2/THBS1/EGFR/INS/CRKL/FGF5/FGF7/PDGFC/KDR/FGF20/PDGFRB/ANGPT2/ANGPT1/EGF/HGF/VEGFC/IGF1/NGF/PGF/VEGFA/FGF17/KIT/MET/EPHA2 |
| 19 | hsa04066 | HIF-1 signaling pathway | 19 | 2.46E-07 | FLT1/ANGPT2/ANGPT1/IFNGR1/EPO/NOS3/EGF/SERPINE1/IGF1/ENO2/EGFR/INS/VEGFA/IL6/IFNG/ERBB2/TIMP1/LTBR/IL6R |
| 20 | hsa05418 | Fluid shear stress and atherosclerosis | 21 | 5.35E-07 | NQO1/BMPR2/SDC4/IL1R1/NOS3/MMP2/PDGFB/PDGFA/TNF/ICAM1/TNFRSF1A/VEGFA/IL1A/IFNG/CTSL/GPC1/KDR/CCL2/SDC1/ITGAV/BMPR1A |
| 21 | hsa05218 | Melanoma | 15 | 7.08E-07 | PDGFRB/EGF/HGF/PDGFB/PDGFA/IGF1/FGF2/EGFR/FGF5/FGF17/FGF7/PDGFC/MDM2/FGF20/MET |
| 22 | hsa04510 | Focal adhesion | 25 | 1.31E-06 | FLT1/LAMA4/PDGFB/XIAP/PDGFA/THBS2/THBS1/EGFR/CRKL/COMP/ERBB2/PDGFC/KDR/SPP1/ITGAV/PDGFRB/EGF/HGF/ITGA1/VEGFC/IGF1/PGF/VEGFA/ITGA5/MET |
| 23 | hsa04062 | Chemokine signaling pathway | 23 | 7.21E-06 | CXCL6/CCL23/CCL11/CXCL8/CXCL1/CXCL3/CXCL2/CXCL5/CX3CL1/CXCL16/CRKL/FGR/CXCL10/CXCL11/CCL8/CCL7/GRK5/CCL5/CCL4/CCL3/CCL2/CCL19/CCL18 |
| 24 | hsa04672 | Intestinal immune network for IgA production | 11 | 1.81E-05 | IL10/IL15RA/IL6/TGFB1/IL5/IL15/CD28/TNFRSF13C/LTBR/IL2/ICOSLG |
| 25 | hsa05219 | Bladder cancer | 10 | 2.57E-05 | CXCL8/MMP1/EGF/MMP2/ERBB2/MDM2/THBS1/EGFR/TYMP/VEGFA |
| 26 | hsa04933 | AGE-RAGE signaling pathway in diabetic complications | 15 | 3.81E-05 | TGFB2/TGFB1/CXCL8/NOS3/MMP2/SERPINE1/VEGFC/F3/TNF/AGT/ICAM1/VEGFA/IL1A/IL6/CCL2 |
| 27 | hsa05146 | Amoebiasis | 15 | 4.77E-05 | IL10/TGFB2/TGFB1/CXCL8/CSF2/IL1R1/LAMA4/CXCL1/CXCL3/CXCL2/TNF/SERPINB6/IL6/IFNG/CD14 |
| 28 | hsa05142 | Chagas disease | 15 | 4.77E-05 | IL10/TGFB2/TGFB1/CXCL8/IFNGR1/SERPINE1/TNF/IL2/TNFRSF1A/IL6/IFNG/CCL5/CCL3/FAS/CCL2 |
| 29 | hsa04020 | Calcium signaling pathway | 24 | 8.10E-05 | PDGFRB/RET/FLT1/NOS3/EGF/HGF/NTRK3/TNNC1/MST1/PDGFB/VEGFC/PDGFA/NGF/FGF2/EGFR/VEGFA/FGF5/FGF17/FGF7/PDGFC/ERBB2/FGF20/KDR/MET |
| 30 | hsa05215 | Prostate cancer | 14 | 1.14E-04 | PDGFRB/EGF/MMP3/PDGFB/PDGFA/KLK3/IGF1/EGFR/INS/SPINT1/PLAU/PDGFC/ERBB2/MDM2 |
| 31 | hsa05320 | Autoimmune thyroid disease | 10 | 2.13E-04 | IL10/TPO/TG/IL5/CD28/FAS/CTLA4/CGA/TSHB/IL2 |
| 32 | hsa04350 | TGF-beta signaling pathway | 13 | 3.30E-04 | TGFB2/TGFB1/BMPR2/FST/BMP8A/INHBA/RGMB/THBS1/TNF/DCN/BMP5/IFNG/BMPR1A |
| 33 | hsa04380 | Osteoclast differentiation | 15 | 5.48E-04 | IFNAR2/TGFB2/SPI1/TGFB1/IL1R1/IFNGR1/SIRPG/TREM2/TNFRSF11B/TNF/TNFRSF1A/IL1A/IFNG/FOSB/ACP5 |
| 34 | hsa04142 | Lysosome | 15 | 7.48E-04 | SCARB2/SORT1/FUCA1/CTSV/CTSS/IGF2R/LAMP1/CTSL/LAMP2/CTSH/ACP5/TPP1/GUSB/CTSB/LGMN |
| 35 | hsa05133 | Pertussis | 11 | 8.08E-04 | IL10/IL1A/CXCL6/IL6/CASP7/CXCL8/IL23A/CD14/ITGA5/TNF/CXCL5 |
| 36 | hsa04659 | Th17 cell differentiation | 13 | 0.00117252 | IL21/TGFB1/IL4R/IL1R1/IFNGR1/IL23R/IL1RAP/IL2/IL6/IFNG/IL23A/IL6ST/IL6R |
| 37 | hsa05167 | Kaposi sarcoma-associated herpesvirus infection | 18 | 0.00185281 | IFNAR2/CXCL8/ANGPT2/CSF2/IFNGR1/PDGFB/CXCL1/CXCL3/FGF2/CXCL2/ICAM1/TNFRSF1A/VEGFA/IL6/CD200R1/UBB/FAS/IL6ST |
| 38 | hsa05417 | Lipid and atherosclerosis | 19 | 0.00226463 | CXCL8/NOS3/MMP1/MMP3/CXCL1/CXCL3/CXCL2/TNF/ICAM1/TNFRSF1A/CASP7/IL6/CCL5/CCL3/FAS/CCL2/OLR1/CD14/LDLR |
| 39 | hsa04620 | Toll-like receptor signaling pathway | 12 | 0.00276274 | TLR1/IFNAR2/CXCL10/IL6/CXCL11/CXCL8/CCL5/CCL4/SPP1/CCL3/CD14/TNF |
| 40 | hsa04660 | T cell receptor signaling pathway | 12 | 0.00276274 | IL10/CSF2/IL5/IFNG/GRAP2/CD28/CTLA4/PTPN6/BCL10/TNF/IL2/NCK1 |
| 41 | hsa04210 | Apoptosis | 14 | 0.00293497 | DFFA/HTRA2/XIAP/CSF2RB/CTSV/NGF/TNF/CTSS/TNFRSF1A/CASP7/CTSL/FAS/CTSH/CTSB |
| 42 | hsa05152 | Tuberculosis | 16 | 0.00540082 | IL10/TGFB2/TGFB1/IFNGR1/BCL10/TNF/CTSS/TNFRSF1A/TLR1/IL1A/IL6/IFNG/LAMP1/IL23A/LAMP2/CD14 |
| 43 | hsa04610 | Complement and coagulation cascades | 10 | 0.00650024 | CFD/F7/F10/PLAU/SERPINE1/PLAUR/PLG/MASP1/TFPI/F3 |
| 44 | hsa05166 | Human T-cell leukemia virus 1 infection | 18 | 0.00734633 | IL15RA/TGFB2/SPI1/TGFB1/MMP7/CSF2/IL1R1/IL15/XIAP/TNFRSF13C/TNF/IL2/ICAM1/TNFRSF1A/IL6/LTA/LTBR/B2M |
| 45 | hsa04512 | ECM-receptor interaction | 10 | 0.00813199 | COMP/SDC4/LAMA4/ITGA1/SPP1/SDC1/ITGAV/ITGA5/THBS2/THBS1 |
| 46 | hsa05164 | Influenza A | 15 | 0.0082345 | IFNAR2/PRSS1/CXCL8/IFNGR1/PLG/TNF/ICAM1/TNFRSF1A/IL1A/CXCL10/IL6/IFNG/CCL5/FAS/CCL2 |
| 47 | hsa05163 | Human cytomegalovirus infection | 18 | 0.00835279 | CXCL8/IL1R1/TNF/EGFR/CX3CL1/CRKL/TNFRSF1A/VEGFA/IL6/CCL5/CCL4/MDM2/CCL3/FAS/CCL2/ITGAV/IL6R/B2M |
| 48 | hsa05410 | Hypertrophic cardiomyopathy | 10 | 0.00938162 | IL6/TGFB2/TGFB1/TNNC1/ITGA1/ITGAV/IGF1/ITGA5/TNF/AGT |
| 49 | hsa04650 | Natural killer cell mediated cytotoxicity | 12 | 0.01162504 | IFNAR2/KLRK1/CSF2/IFNG/IFNGR1/SH2D1A/ICAM2/FAS/PTPN6/ULBP2/TNF/ICAM1 |
| 50 | hsa05226 | Gastric cancer | 13 | 0.01567329 | TGFB2/TGFB1/EGF/HGF/FGF2/EGFR/LRP6/FGF5/FGF17/FGF7/ERBB2/FGF20/MET |
| 51 | hsa05171 | Coronavirus disease - COVID-19 | 17 | 0.02289741 | CFD/IFNAR2/CSF3/CXCL8/CSF2/MMP1/MMP3/TNF/EGFR/IL2/TNFRSF1A/CXCL10/IL6/CCL2/MASP1/IL6ST/IL6R |
| 52 | hsa04810 | Regulation of actin cytoskeleton | 16 | 0.02747735 | PDGFRB/EGF/ITGA1/PDGFB/PDGFA/FGF2/EGFR/INS/CRKL/FGF5/FGF17/FGF7/PDGFC/FGF20/ITGAV/ITGA5 |
| 53 | hsa04360 | Axon guidance | 14 | 0.0297171 | EPHB6/SEMA6B/ROBO3/SEMA7A/BMPR2/RYK/UNC5C/EFNB3/BOC/EPHA1/MET/EPHB4/EPHA2/NCK1 |
| 54 | hsa05224 | Breast cancer | 12 | 0.0325724 | FGF17/FGF5/NOTCH3/FGF7/EGF/ERBB2/KIT/FGF20/IGF1/FGF2/EGFR/LRP6 |
| 55 | hsa05145 | Toxoplasmosis | 10 | 0.03432345 | IL10/TGFB2/TGFB1/IFNG/IFNGR1/LAMA4/XIAP/TNF/LDLR/TNFRSF1A |
| 56 | hsa05202 | Transcriptional misregulation in cancer | 14 | 0.04447647 | SPI1/FLT1/CXCL8/CSF2/IGFBP3/MMP3/PDGFA/IGF1/IL6/SPINT1/PLAU/MDM2/CD14/MET |
| 57 | hsa04068 | FoxO signaling pathway | 10 | 0.07758086 | IL10/IL6/TGFB2/TGFB1/EGF/MDM2/IGF1/IL7R/EGFR/INS |
| 58 | hsa04145 | Phagosome | 11 | 0.08171107 | COMP/LAMP1/CTSL/LAMP2/OLR1/ITGAV/CD14/ITGA5/THBS2/THBS1/CTSS |
