# Supplementary figures and images for: Menstrual blood-derived endometrial stem cells alleviate neuroinflammation by modulating M1/M2 polarization in cell and rat Parkinson’s disease models
Source: Stem Cell Res Ther. 2023 Apr 13;14:85. doi: 10.1186/s13287-023-03330-7 (PMC10099022; doi:10.1186/s13287-023-03330-7)

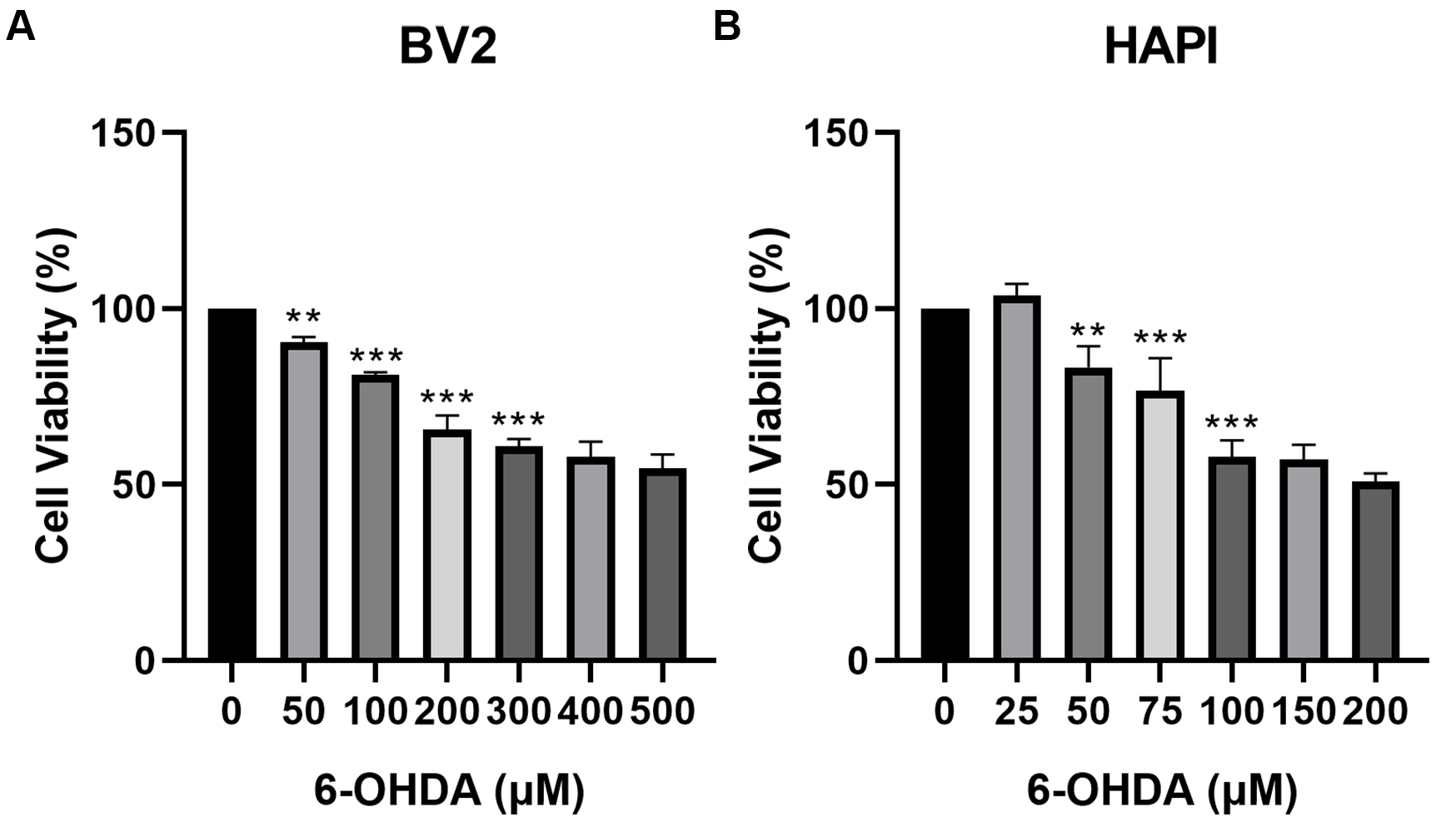

Supplement: Supplementary file 2 — Additional file 2: Fig. S1. The cell viability of BV2 and HAPI cells. A: BV2 cells were treated with various concentrations of 6-OHDA (0, 50, 100, 200, 300, 400 and 500 µM) for 24 h. B: HAPI cells were treated with various concentrations of 6-OHDA (0, 25, 50, 75, 100, 150, and 200 µM) for 24 h. Cell viability was measured using PrestoBlue reagent. [file 13287_2023_3330_MOESM2_ESM.tif]

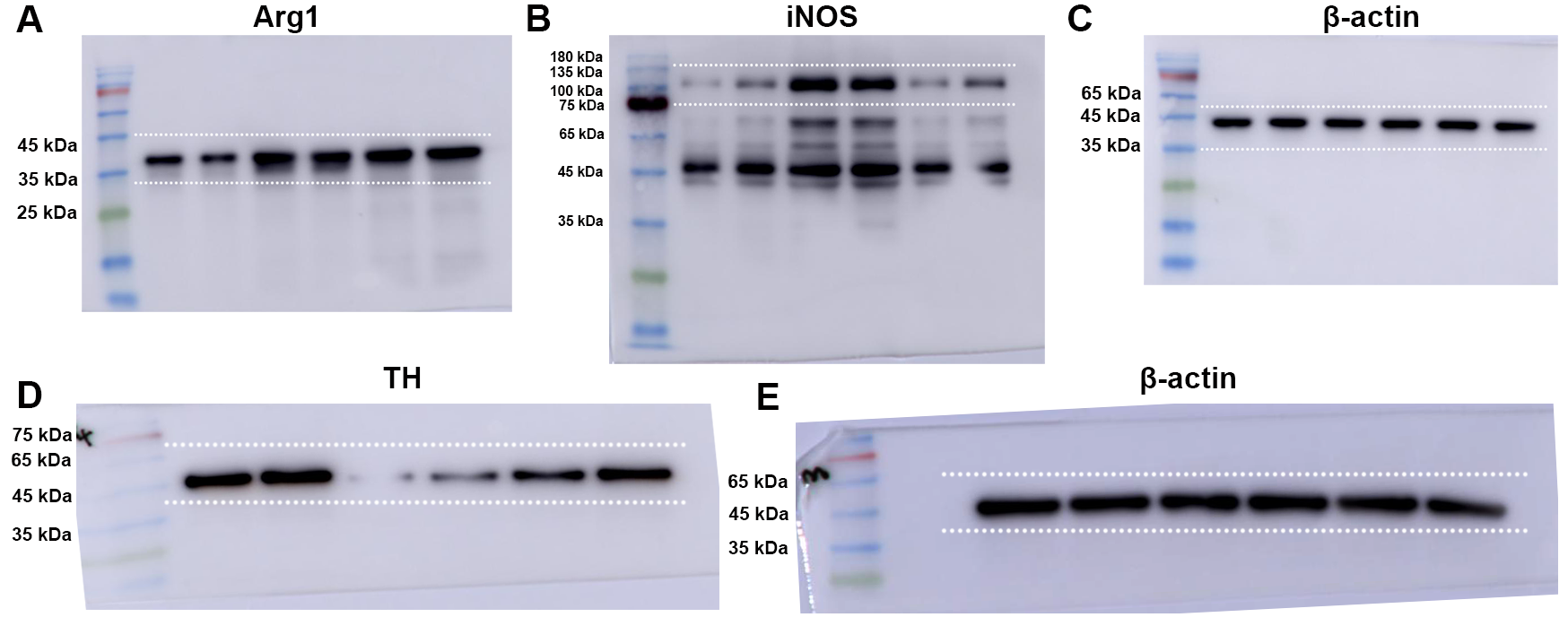

Supplement: Supplementary file 3 — Additional file 3: Fig. S2. Uncropped WB images of Arg1, iNOS, TH and β-actin. A: WB result of Arg1 (36 kDa). B: WB result of iNOS (110-130 kDa, 65-70 kDa). C: WB result of β-actin (43 kDa). D: WB result of TH (57 kDa). E: WB result of β-actin (43 kDa). Dashed lines indicated where they were cropped to make Fig.6C and Fig.4J. [file 13287_2023_3330_MOESM3_ESM.tif]
